# Surveillance of zoonotic and production-limiting pathogens in invasive free-ranging wild pigs in Alberta, Canada

**DOI:** 10.1186/s12917-026-05793-1

**Published:** 2026-08-05

**Authors:** Oshin Ley-Garcia, Chunu Mainali, Frank van der Meer, Davor Ojkic, Carolina Malgarin, Bryna Turk, Sylvia L. Checkley, Hannah McKenzie, Mathieu Pruvot

**Affiliations:** 1 Faculty of Veterinary Medicine, University of Calgary, Calgary, AB T2N 4N1 Canada; 2 Government of Alberta, Edmonton, AB T5K 1E7 Canada; 3 Animal Health Laboratory, Laboratory Services Division, University of Guelph, Guelph, ON Canada; 4 Veterinary Services, Merck Animal Health, Kirkland, QC Canada; 5 Wildlife Conservation Society, Health Program, Bronx, NY USA

**Keywords:** Wild pigs, Reservoirs, Pathogens, Risk, Porcine circovirus, Rotavirus, Parasites

## Abstract

**Background:**

The presence of wild pigs across the prairie provinces in Canada, coupled with their potential role as reservoirs for economically significant livestock pathogens, has intensified the need for surveillance. This study provides an overview of swine production-limiting diseases circulating within the invasive wild pig population in Alberta.

**Results:**

Between 2018 and 2024, 331 individuals were captured and tested for 17 pathogens. Antibodies to *Erysipelothrix rhusiopathiae* (120/120; 100%), porcine circovirus 2 (PCV2, 38/120; 31.7%), *Mycoplasma hyopneumoniae* (*Mhyo*, 2/96; 2.1%), and porcine reproductive and respiratory syndrome virus (PRRSV, 1/96; 1.0%) were detected. Gastrointestinal parasites were identified for *Eimeria* spp. (76/192; 39.6%); *Metastrongylus* spp. (49/192; 25.5%); *Trichuris* spp. (11/192; 5.7%); *Ascaris* spp. (11/192; 5.7%); and *Strongyloides* spp. (8/192; 4.2%). Molecular diagnostics confirmed infection with porcine circovirus 1 (PCV1, 1/331; 0.3%), PCV2 (109/331; 32.9%), porcine circovirus 3 (PCV3, 90/331; 27.2%), rotavirus A (RVA, 21/92; 22.8%), rotavirus C (RVC, 5/92; 5.4%), porcine parvovirus (PPV, 18/101; 17.8%), and *Salmonella* spp. (2/72; 2.8%). Porcine deltacoronavirus (PDCoV), porcine epidemic diarrhea virus (PEDV), Seneca virus A (SVA), transmissible gastroenteritis virus (TGEV), influenza A virus (IAV), and rotavirus B (RVB) were not detected. Age was significantly associated with PCV2 and PCV3 infection, with mature individuals exhibiting a higher risk (*p* < 0.001) of being infected. Co-detection of pathogens was common, with 55.9% (38/68) of wild pigs testing positive for two or more pathogens.

**Conclusion:**

Overall, wild pigs were infected with pathogens that are significant to the porcine production industry and public health, demonstrating the potential risk of transmission at the wild and domestic pig interface, and highlighting the importance of continued surveillance from a One Health perspective.

**Supplementary Information:**

The online version contains supplementary material available at https://doi.org/10.1186/s12917-026-05793-1.

## Background

Wild pigs *(Sus scrofa)*, also referred to as wild boar, wild suid, wild/feral swine, wild/feral hog, or feral pig, are recognized as one of the most detrimental invasive species in North America, contributing to significant agricultural damages, environmental degradation, and biodiversity loss [1]. Wild pigs were introduced to Canada in the 1980s and 1990s as a component of agricultural diversification programs [2]. Subsequent escapes and releases from wild pig farms contributed to the establishment of free-ranging populations, which have since expanded across the prairie provinces [3]. Their persistence has increased concerns regarding the associated risk of infectious disease transmission at the wild-domestic pig interface [4].

Wild pigs pose a major disease infection risk, as 87% of the swine pathogens listed by the World Organization for Animal Health (WOAH) are transmissible to other species, including wildlife, livestock, and humans [5]. This risk is particularly concerning in areas where wild and domestic pigs (*Sus scrofa domesticus*) coexist, as their close genetic relationship facilitates efficient cross-host pathogen transmission [6].

Alberta produces a substantial amount of pork, with over 2.87 million pigs marketed annually, supported by both intensive commercial farms and small-scale operations [7]. The 2023 agricultural census recorded 1,100 pig-holding operations in Alberta, of which 278 (25.3%) operated as commercial production systems, while 819 (74.5%) were smallholder or backyard farms [8]. Given that the province is dominated by small-scale operations, sustained wild pig surveillance is particularly important to understand the potential for pathogen circulation, as these small-scale operations may provide more opportunities for interaction between wild and domestic pigs [9].

Several production-limiting pathogens are endemic in Canadian pig populations, including porcine reproductive and respiratory syndrome virus (PRRSV) [10], porcine circoviruses (PCVs) [11], *Mycoplasma hyopneumoniae (Mhyo*) [12], porcine parvovirus (PPV) [13], influenza A virus (IAV) [14], rotaviruses (RVs) [15], *Actinobacillus pleuropneumoniae* [16], and *Erysipelothrix rhusiopathiae* [17]. Additionally, outbreaks of porcine deltacoronavirus (PDCoV), porcine epidemic diarrhea virus (PEDV), transmissible gastroenteritis virus (TGEV), and Seneca virus A (SVA) have been reported in Canadian swine farms [18–20]. These viruses pose a substantial economic burden [21], while SVA can trigger foreign animal disease investigations due to its clinical similarity to foot-and-mouth disease (FMD) and other vesicular diseases [22].

In addition to their production impacts, several of these pathogens, including IAV, RVs, *Erysipelothrix rhusiopathiae*, and *Salmonella* spp., also have zoonotic relevance [14, 23–26].

Evidence from the United States and Saskatchewan indicates that wild pigs can act as reservoirs for a wide range of production-limiting and zoonotic pathogens [27, 28].

However, the infection status of Alberta’s wild pig population remains unknown. Given this knowledge gap, this study aimed to investigate the occurrence of swine production-limiting diseases in free-ranging invasive wild pigs in Alberta, Canada.

## Materials and methods

### Field sampling

**Fig. 1:**
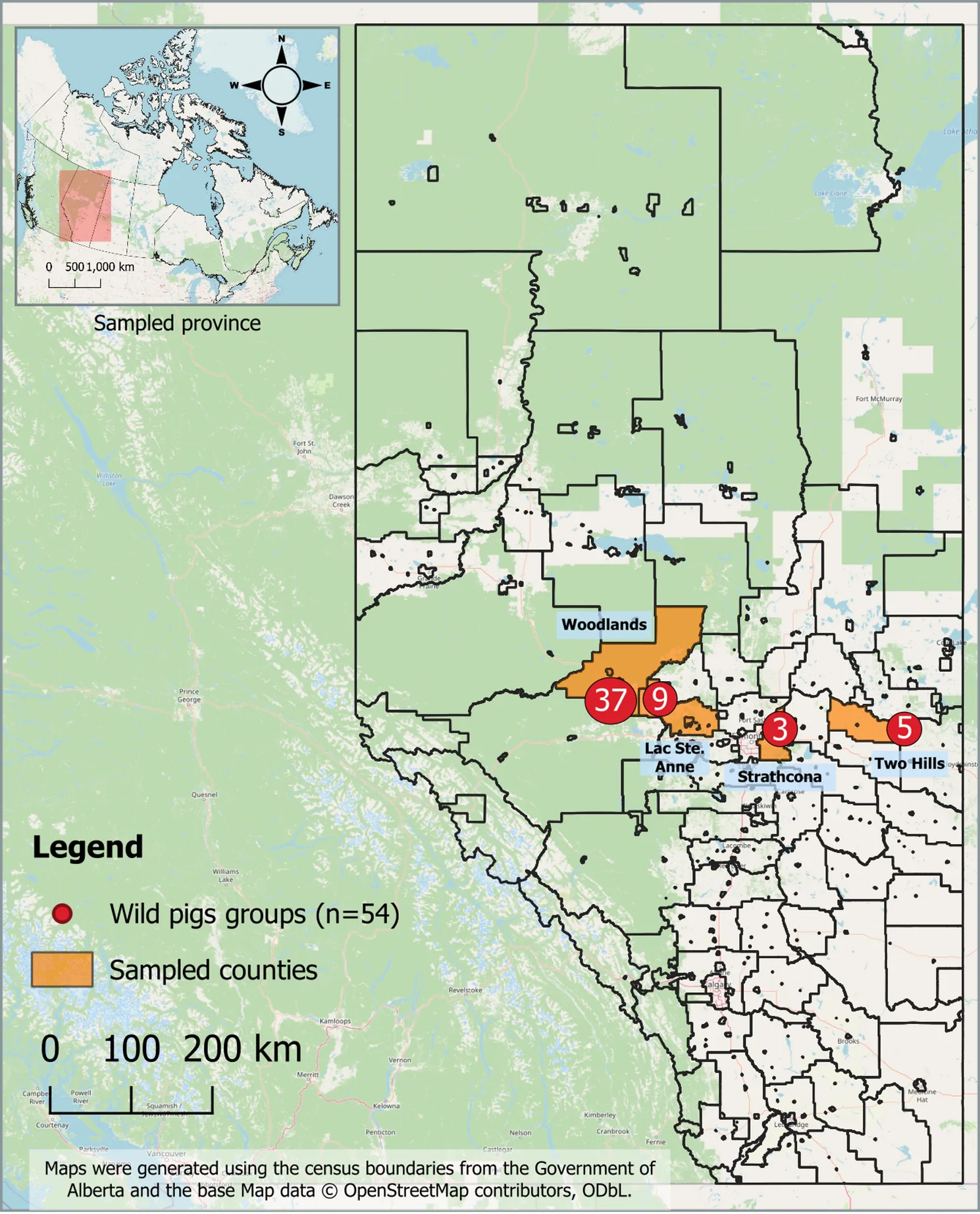
Study area and locations of captured wild pig groups in Alberta, Canada, during the period 2018–2024. The map shows the delineated census subdivision (counties) in Alberta from which a total of 54 wild pig groups were retrieved: Woodlands, Lac Ste. Anne, Strathcona, and Two Hills. Trapping locations are displayed as red circles, with circle size indicating the number of wild pig groups captured at each location. Maps were projected using the Coordinate Reference System (CRS): WGS 84 / Pseudo-Mercator EPSG: 3857. Spatial data preparation and mapping were completed using QGIS *v3.34.12*

As part of the Alberta Wild Boar Control Program led by the Government of Alberta, wild pigs were captured across the province (Fig. 1) from 2018 to 2024, using whole-sounder trapping techniques and euthanized. Following euthanasia, 3 mL of blood was collected from the heart (BD Vacutainer, ThermoFisher Scientific, Waltham MA, USA). Full carcasses were submitted to the *Animal Health Section*,* Animal Health and Assurance Branch*,* Primary Agriculture Division*,* Alberta Agriculture and Irrigation (AGI)* (Edmonton, AB, Canada). During necropsy, weight before evisceration, body condition score (BCS), age classification based on the presence of stripes (juvenile, mature), sex (male, female), pregnancy status and number of fetuses in pregnant females, and gross lesions of major organs were recorded.

### Antibody testing

At the laboratory, blood samples were centrifuged (Eppendorf centrifuge, Sigma-Aldrich, Oakville, ON, CA) at 3000 rpm for 15 min to separate serum, which was then aliquoted. Processed serum (*n* = 120) and tissue samples (*n* = 331) were stored at -80 °C. Archived samples were subsequently transferred to the University of Calgary for diagnostic analysis.

Serum samples were first screened using a commercially available indirect enzyme-linked immunosorbent assay (ELISA) kit for the detection of IgG antibodies to PCV2 (Swinecheck PCV2, Biovet, St. Hyacinthe, QC, Canada) following the manufacturer’s instructions.

Samples were also tested for antibodies against PRRSV types 1 (EU) and 2 (NA), and *Mycoplasma hyopneumoniae* (Biovet Veterinary Diagnostic Services, St. Hyacinthe, QC, Canada) using a microsphere-based multiplex fluorescent immunoassay (Swinecheck MP PRRSV 1 & 2, *M. hyopneumoniae*).

Additionally, an adapted commercial ELISA for the detection of porcine *E. rhusiopathiae* was performed. This assay followed the rSpaA415-based method described by Mavrot et al. [29], with adaptations including the use of in-house–produced recombinant SpaA antigen, a customized casein/BSA/Tween blocking buffer, optimized sample dilution (1:10), and Protein A/G–HRP–based detection.

### Detection of bacteria

The bacteriological testing of *Salmonella* spp. was conducted at the Animal Health Section, Animal Health and Assurance Branch, Primary Agriculture Division, Alberta Agriculture and Irrigation (AGI) (Edmonton, AB, Canada). Supplementary Material S1 provides additional details on the *Salmonella* spp. diagnostic procedures.

### Detection of viruses

Nucleic acid was extracted following commercial protocols (MagMAX Viral RNA Isolation Kit, MagMAX Core Nucleic Acid Purification Kit, MagneSil KF Genomic System Kit, and Qiagen DNeasy Blood & Tissue Kit). The extraction kits, sample types, and corresponding target pathogens for each assay are summarized in Supplementary Table S1.

For simplicity, all amplification assays are referred to as ‘real-time PCR’ or ‘conventional PCR’ throughout the text. Detailed protocols for all PCR conditions, including sequencing primers and probes, reaction mixes, and thermocycling parameters, are provided in the Supplementary Materials (Table S2-S9).

Real-time PCR tests for Seneca virus A (SVA), influenza A virus (IAV), porcine epidemic diarrhea virus (PEDV), porcine deltacoronavirus (PDCoV), and transmissible gastroenteritis virus (TGEV), were conducted at AGI (Edmonton, AB, Canada).

Real-time PCR tests for porcine circovirus (PCV1, PCV2, and PCV3), and porcine parvovirus (PVV) were done at the University of Guelph AAVLD-accredited Animal Health Laboratory (Guelph, ON, Canada).

Multiplex real-time PCR tests for rotaviruses (RVs) subtype RVA, RVB, and RVC were performed at the Diagnostic Services Unit (DSU) (Calgary, AB, Canada).

### Detection of parasites

Fecal samples were processed using a modified Wisconsin Sugar Centrifugal-Flotation technique [30] to detect gastrointestinal parasites. Briefly, 3 g of feces were homogenized with 10 mL of Sheather’s Sugar Solution (ThermoFisher Scientific, Waltham, MA, USA) in a disposable cup and stirred until uniformly suspended. The mixture was strained through a single layer of cheesecloth into a second cup to remove debris. The filtrate was transferred into a centrifuge tube, filled to approximately 2 cm from the rim, and centrifuged at 500 × g for 10 min. After centrifugation, the tube was topped off with additional sugar solution to form a convex meniscus, and a coverslip was carefully placed on top. Tubes were left undisturbed for 10 min to allow parasite eggs, cysts, and oocysts to adhere to the coverslip. The coverslip was then removed by gently lifting it straight upward and placed onto a glass microscope slide. Preparations were examined under a microscope (Olympus BX53, Olympus Corporation, Tokyo, Japan) at 100× total magnification. Parasite oocysts/eggs were counted and identified morphologically based on size, shape, and internal structures, following the description by Otranto and Wall [31].

### Statistical analysis

All the analyses were performed with *R Software v4.5.2* [32].

Testing was conducted opportunistically, and the final sample size tested was primarily determined by sample availability and by the number of samples submitted in each capture year. The yearly distribution of samples tested is provided in Supplementary Table S10. Because the number of samples tested varied across pathogens, we conducted a post-hoc power analysis to estimate the lowest prevalence that could be reliably detected for each pathogen given its final sample size. Post-hoc minimum detectable prevalence was estimated using the *epiR* package, which implements the standard binomial detection-probability framework for surveillance design. Exact 95% binomial confidence intervals were obtained using the *binom* package. Detailed post-hoc results are provided in the Supplementary Materials (Table S11).

The frequency of occurrence of each pathogen, overall and disaggregated by biological and geographic variables, and 95% confidence intervals (CI) were estimated with the *epiR* package. Data visualization was conducted using the *ggplot2* package.

The frequency of pathogen co-detection was estimated using a restricted dataset consisting of wild pigs sampled between 2022 and 2024 that had complete molecular (PCR, detection of pathogen genetic material) and coprological results (detection of parasite eggs or oocysts). Serological results were excluded because they reflect historical exposure rather than active infection, and diagnostic outcomes that were incomplete or not fixed were removed. Co-detection was defined as the presence of two or more pathogen-specific markers detected in the same individual. The frequency of pathogen co-detection was calculated as the proportion of animals testing positive for multiple pathogens (e.g., ≥ 2 pathogens) divided by the total number of animals meeting these criteria (*n* = 68). To evaluate whether specific pathogens detections occurred together more often than expected by chance, pairwise associations among the dichotomous (binary) pathogen detection variables were assessed using the non-parametric *Phi coefficient* test. Pathogens were included in this analysis when: (i) they were tested in at least 92 animals, and (ii) they had a minimum of four positive detections, ensuring adequate 2 × 2 contingency tables for the *Phi test*. Only pathogens detected by molecular (PCR) and coprological methods were included in this analysis. Because conducting multiple tests increases the likelihood of false-positive results, p-values were adjusted using the Benjamini–Hochberg false discovery rate (FDR) method. Correlation matrices were generated with the *vcd* package, and statistical significance was determined based on the FDR‑adjusted p‑values.

Univariate mixed-effects logistic regression models were built to assess risk factors for PCV2, PCV3, PPV, and RVA molecular detection, as well as *Eimeria* spp. and *Metastrongylus* spp. coprological detection, using the test results (positive or negative) as the binary outcome (dependent variable). These pathogens were selected for regression analysis because they had sufficient numbers of positive detections and adequate samples sizes to support stable parameter estimation and model convergence. The group of capture was included as a random effect to account for clustering of samples within wild pig groups. Candidate independent categorical variables included sex, age, pregnancy status, county of capture, season, and year/period. Candidate independent continuous variables included weight, wild pigs group size, density of domestic pigs (commercial and small-scale), minimum distance to the nearest domestic pig farms (commercial and small-scale), and minimum distance to the nearest historical wild pig farms. Details on the derivation and calculation of the spatial variables are provided in the Supplementary Methods (Table S12). Statistical significance was set at alpha = 0.05. Model performance was assessed using Akaike’s Information Criteria (AIC), and the Area Under the Receiver Operating Characteristic Curve (AUC-ROC). All generalized linear mixed models (GLMM) were fitted using the *lme4* package, and AUC-ROC values were computed using the *pROC* package.

## Results

### Demographic and study population attributes

A total of 331 wild pigs were captured from 2018 to 2024. Table 1 summarizes the biological and spatiotemporal characteristics of the sampled animals. Higher frequencies were observed in the categories of males and mature individuals, and those with a normal weight condition. Most captures occurred during the winter season and in Woodland County. The highest number of captures was recorded in 2021. Among the 164 sampled females, 30 were pregnant. Juvenile wild pigs weighed on average 11.44 kg, with a normal weight range of 4.79–18.01 kg (SD = 6.61 kg). Mature animals averaged 55.71 kg (range: 30.2–81.2 kg, SD = 25.5). Wild pigs were often captured in social groups, with an average group size of 6 animals (range: 1–32).

**Table 1:** Biological and spatial-temporal variables assessed in wild pigs captured in Alberta, Canada (2018–2024), and their frequency distribution by category

Variable	Category	Frequency of observation (%)	Fisher’s 95% CI ^a^
Sex (*n* = 331)	Female	164 (49.5%)	44.0–55.1
	Male	167 (50.5%)	44.9–56.0
Age (*n* = 331) ^b^	Juvenile	121 (36.6%)	31.4–42.0
	Mature	210 (63.4%)	58.0–68.7
Pregnancy Status (*n* = 117) ^c^	Pregnant	30 (25.6%)	18.0–34.5
	No Pregnant	87 (74.4%)	65.5–82.0
Juvenile Weight (*n* = 121) ^d^	Underweight	2 (1.7%)	0.2–5.8
	Normal weight	100 (82.7%)	74.7–88.9
	Overweight	19 (15.6%)	9.7–23.4
Mature Weight (*n* = 209) ^d, e^	Underweight	49 (23.4%)	17.9–29.8
	Normal weight	124 (59.3%)	52.39–66.1
	Overweight	36 (17.3%)	12.4–23.0
BCS (*n* = 303) ^f^	1 (Emaciated)	3 (1%)	0.24–2.9
	2 (Thin)	90 (29.7%)	24.64–35.2
	3 (Ideal)	183 (60.4%)	54.64–65.9
	4 (Rounded)	23 (7.6%)	4.9–11.2
	5 (Obese)	4 (1.3%)	0.4–3.4
County of captured (*n* = 331) ^g^	WL	222 (67.1%)	61.7–72.1
	LSA	77 (23.3%)	18.8–28.2
	TH	21 (6.3%)	4.0–9.5
	SC	11 (3.3%)	1.7–5.9
Season of collection (*n* = 331) ^h^	Winter	149 (45.0%)	39.6–50.6
	Spring	116 (35.0%)	29.9–40.5
	Summer	33 (10.0%)	6.7–13.7
	Fall	33 (10.0%)	6.7–13.7
Year of collection (*n* = 331)	2018	18 (5.4%)	3.3–8.5
	2019	32 (9.7%)	6.7–13.4
	2020	14 (4.2%)	2.3–7.0
	2021	100 (30.2%)	25.3–35.5
	2022	79 (23.9%)	19.4–28.4
	2023	25 (7.6%)	5.0–11.0
	2024	63 (19.0%)	15.0–23.7

^a^ CI: Confidence Interval

^b^ Juvenile age was based on the visible presence of stripes

^c^ Data was not available for all wild sows; 47 females were not classified

^d^ Weight: Animals with weights (kg) within the mean range of mean ± 1 standard deviation (SD) for their age category were considered to have normal weight. Those under or above this range were classified as underweight or overweight, respectively

^e^ Data for one wild pig was missing; therefore, one individual was not classified

^f^ BSC: Body condition score, data were not available for all wild pigs; 28 individuals were not classified

^g^ County, WL: Woodland, LSA: Lac Ste. Anne, TH: Two Hills, and SC: Strathcona

^h^ Season of collection was defined using astronomical start dates: Winter (December 21), Spring (March 20), Summer (June 20), and Fall/Autumn (September 22)

### Swine pathogen detection

Results from serological, bacteriological, parasitological, and molecular testing are summarized in Table 2. Overall, *E. rhusiopathiae*, PCV2, PCV3, *Eimeria* spp., and RVA were the most commonly identified pathogens. Antibodies against *E. rhusiopathiae*, PCV2, *Mhyo*, and PRRSV were detected by ELISA. A single wild pig was seropositive for PRRSV type 1 (European genotype), with an S/P value of 0.36, slightly above the manufacturer’s positivity threshold (S/*P* > 0.30). The two Salmonella-positive samples were confirmed as *Salmonella enterica* subsp. *diarizonae* serotype 61: k:1,5, (7) (SED) and *Salmonella enterica* serotype Montevideo (MV) by agglutination test. All surveyed wild pigs were negative by PCR for IAV, SVA and *Coronaviridae* (PDCoV, PEDV, and TGEV). Images of oocysts of *Eimeria* spp. and eggs of *Metastrongylus* spp., *Trichuris* spp., and *Ascaris* spp. identified during coprological examination are presented in Supplementary Information (Fig. S1).

**Table 2:** Overview of target pathogen detected in captured wild pigs of Alberta during 2018–2024

Pathogen^a^	No. of positive/ Total sample (*n*)	Sample prevalence (%)	95% CI ^b^
Serology			
* E. rhusiopathiae*	120/120	100	97.0–100
PCV2	38/120	31.7	23.5–40.8
* Mhyo*	2/96	2.1	0.3–7.3
PRRSV type 1 (EU)	1/96	1.0	0.03–5.7
PRRSV type 2 (NA)	0/96	-	-
Bacteriology			
* Salmonella* spp.	2/72	2.8	0.34–9.67
Parasitology			
* Eimeria* spp.	76/192	39.6	32.6–46.9
* Metastrongylus* spp.	49/192	25.5	19.5–32.3
* Trichuris* spp.	11/192	5.7	2.9–10.0
* Ascaris* spp.	11/192	5.7	2.9–10.0
* Strongyloides* spp.	8/192	4.2	1.82–8.04
Molecular			
PCV1	1/331	0.3	0.01–1.7
PCV2	109/331	32.9	27.9–38.3
PCV3	90/331	27.2	22.5–32.3
* Salmonella* spp.	2/72	2.8	0.34–9.67
PPV	18/101	17.8	10.91–26.7
PDCoV	0/98	-	-
PEDV	0/98	-	-
SVA	0/96	-	-
TGEV	0/91	-	-
IAV	0/83	**-**	-
RVA	21/92	22.8	14.7–32.8
RVB	0/92	**-**	-
RVC	5/92	5.4	1.79–12.2

^a^ *Erysipelothrix rhusiopathiae*, PCV2: porcine circovirus 2, *Mhyo: Mycoplasma hyopneumoniae*, PRRSV type 1 (EU): porcine reproductive and respiratory syndrome virus type 1 European, PRRSV type 2 (NA): porcine reproductive and respiratory syndrome virus type 2 North America, PCV1: porcine circovirus 1, PCV3: porcine circovirus 3, PPV: porcine parvovirus, PDCoV: porcine deltacoronavirus, PEDV: porcine epidemic diarrhea virus, SVA: Seneca virus A, TGEV: transmissible gastroenteritis virus, IAV: influenza A virus Matrix, RVA: rotavirus A, RVB: rotavirus B, and RVC: rotavirus C

^b^ CI: Confidence Interval

### Frequency of co-detection of target pathogens

Among 68 wild pigs tested for 10 pathogens, 8 animals (11.8%; 95% CI: 5.5.22–21.9) were negative for all pathogens. A total of 22 animals (32.4%; 95% CI: 21.5–44.8) had a single pathogen detected, while 38 animals (55.9%; 95% CI: 43.3–67.9) had co-detection of two or more pathogens.

Co-detections involving two pathogens were most common (18/68; 26.5%; 95% CI: 16.5–38.6), followed by co-detections with three (13/68; 19.1%; 95% CI: 10.6–30.5), and four or more pathogens (7/68; 10.3%; 95% CI: 4.2–20.1), This pattern indicates that co-detections of two pathogens was the most common pattern observed in the wild pig population.

**Fig. 2:**
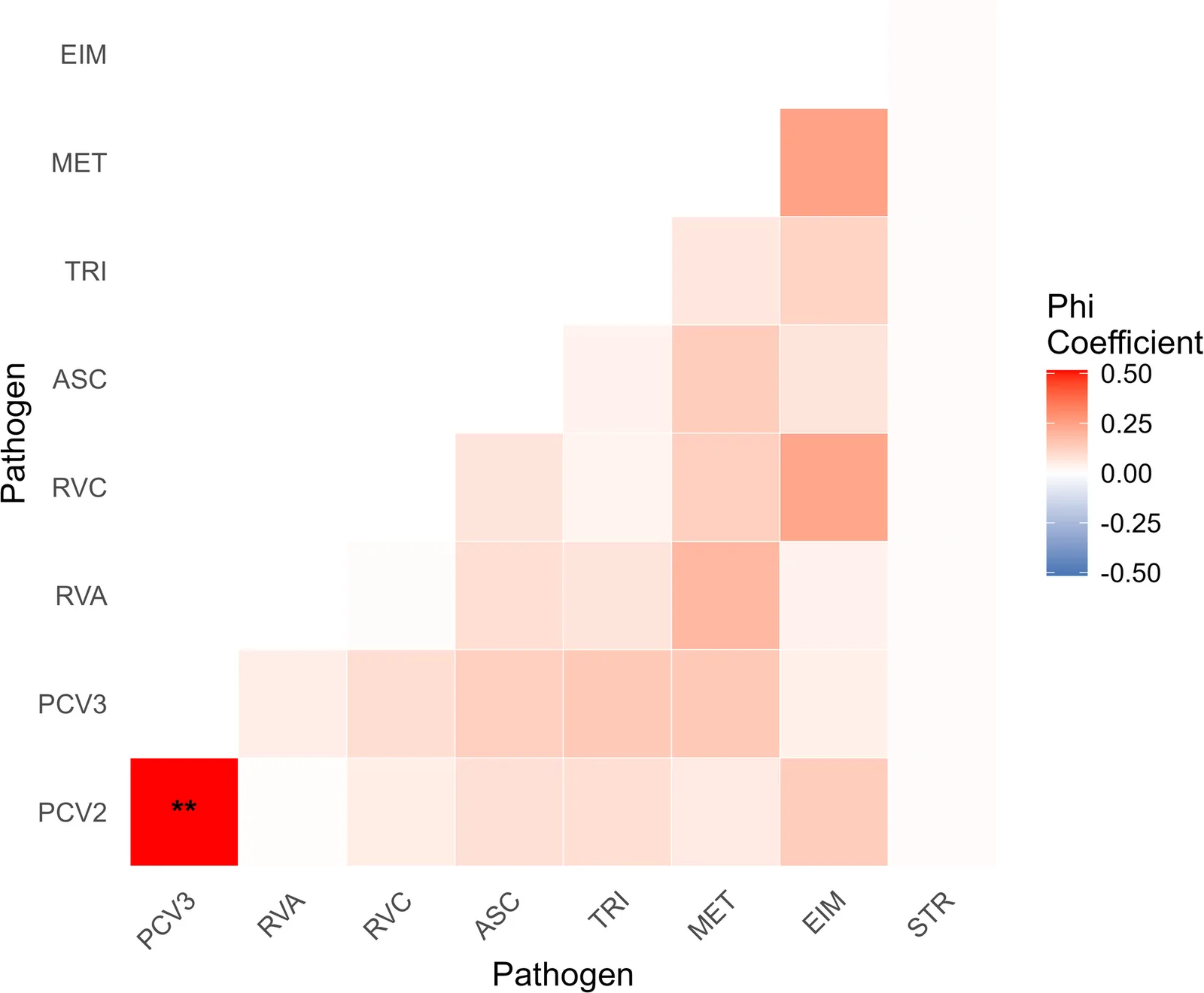
Heat map showing the Phi coefficient matrix between detected pathogens in captured wild pigs (*n* = 68) in Alberta from 2022 to 2024. The Phi coefficient ranges from − 0.50 to 0.50. Red indicates a positive correlation (darker red indicates a stronger correlation); blue indicates a negative correlation. Asterisks denote statistical significance: *p* < 0.01(**). Pathogens included in the analysis were restricted to those tested in ≥ 92 animals and with ≥ 4 positive detections to ensure stable estimates. The Phi coefficient analysis included only pathogens detected by molecular (PCR) and coprological methods. Legend – Pathogens abbreviations: *Eimeria* spp. (EIM), *Metastrongylus* spp. (MET), *Trichuris* spp. (TRI), *Ascaris* spp. (ASC), *Strongyloides* spp. (STR), rotavirus C (RVC), rotavirus A (RVA), porcine circovirus 3 (PCV3), and porcine circovirus 2 (PCV2)

Pairwise associations between pathogen detections were evaluated using the Phi coefficient (Fig. 2). Statistically significant positive association was detected between PCV2 and PCV3 (ϕ = 0.51, *p* < 0.01; 26.5%; 18/68; 95% CI: 16.5–38.6). No other pathogen pairs showed evidence of significant co-detection.

### Analysis of risk factors associated with porcine viruses and parasites

Univariate mixed-effects analysis comparing PCV2-positive (*n* = 109) and PCV2-negative (*n* = 222) wild pigs revealed that the mature category (OR = 31.1; 95% CI: 7.86–123; *p* < 0.001) and weight (OR = 1.06; 95% CI: 1.04–1.08; *p* < 0.001) were significantly associated with an increased risk of PCV2 detection (Tables 3 and 4). No statistically significant associations were found between PCV2 detection and other demographic or geographic variables.

**Table 3:** Univariate mixed-effects logistic regression analysis of risk factors (categorical predictors) associated with PCV2-molecular detection in wild pigs captured in Alberta

Variable	Category	Positive (*n* = 109)	Negative (*n* = 222)	ß ^a^	SE ^b^	OR ^c^	95% CI ^d^	*P*-value
		N (%)	N (%)					
Sex (*n* = 331)	Female (*n* = 164)	59 (36.0)	105 (64.0)	-0.783	0.550	ref	**-**	**-**
	Male (*n* = 167)	50 (29.9)	117 (70.1)	-0.348	0.346	0.71	0.36–1.39	0.314
Age (*n* = 331)	Juvenile (*n* = 121)	20 (16.5)	101 (83.5)	-3.920	0.881	ref	**-**	**-**
	Mature (*n* = 210)	89 (42.4)	121 (57.6)	3.438	0.702	31.1	7.86–123	< 0.001*
Pregnancy Status ^e^ (*n* = 117)	No Pregnant (*n* = 87)	30 (34.5)	57 (65.5)	-0.126	0.830	ref	**-**	**-**
	Pregnant (*n* = 30)	9 (30.0)	21 (70.0)	-0.353	0.932	1.42	0.23–8.84	0.705
County of captured (*n* = 331)	LSA (*n* = 77)	11 (14.3)	66 (85.7)	-2.279	1.164	ref	**-**	**-**
	WL (*n* = 222)	91 (41.0)	131 (59.0)	1.982	1.299	7.26	0.57–92.5	0.127
	TH (*n* = 21)	3 (14.3)	18 (85.7)	-0.684	1.986	0.50	0.01–24.8	0.731
	SC (*n* = 11)	4 (36.4)	7 (63.6)	0.808	2.480	2.24	0.02–290	0.745
Season of collection (*n* = 331)	Fall (*n* = 33)	12 (36.4)	21 (63.6)	0.259	1.412	ref	**-**	**-**
	Winter (*n* = 149)	57 (38.3)	92 (61.7)	-0.801	1.550	0.45	0.02–9.37	0.605
	Spring (*n* = 116)	26 (22.4)	90 (77.6)	-2.449	1.71	0.14	0.00–2.47	0.152
	Summer (*n* = 33)	14 (42.4)	19 (57.6)	-1.232	1.028	0.29	0.04–2.19	0.231
Period (*n* = 331)	2018–2021 (*n =* 164)	60 (36.6)	104 (63.4)	0.202	0.174	ref	**-**	**-**
	2022–2024 (*n* = 167)	49 (29.3)	118 (70.7)	2.840	3.100	2.84	0.33–24.1	0.340

^a^ ß: Estimate

^b^ SE: Standard Error

^c^ OR: Odds Ratio

^d^ CI: Confidence Interval

^e^ Data was not available for all individuals

* Statistical Significance

**Table 4:** Univariate mixed-effects logistic regression analysis of risk factors (continuous predictors) associated with PCV2-molecular detection in wild pigs captured in Alberta

Variable	ß ^a^	SE ^b^	OR ^c^	95% CI ^d^	*P*-value
Weight (*n* = 330) ^e^	0.057	0.011	1.06	1.04–1.08	< 0.001*
Wild pigs group size (*n* = 331)	-0.044	0.065	0.96	0.84–1.09	0.569
Commercial pig density (head/km^2^)	-0.369	0.562	0.69	0.23–2.08	0.511
Small-scale pig density (head/km^2^)	-0.265	0.489	0.77	0.29–2.00	0.587
Distance to commercial pig farms (km)	0.015	0.063	1.02	0.90–1.15	0.813
Distance to small-scale pig farms (km)	0.099	0.265	1.10	0.66–1.86	0.709
Distance to wild pig farms (km)	0.938	0.063	0.94	0.82–1.07	0.344

^a^ ß: Estimate

^b^ SE: Standard Error

^c^ OR: Odds Ratio

^d^ CI: Confidence Interval

^e^ Data was not available for all individuals

* Statistical Significance

Similarly, the mature category was strongly associated with PCV3 infection in wild pigs (OR = 52.2; 95% CI: 13–205; *p* < 0.002), as was weight (OR = 1.05; 95% CI: 1.04–1.08; *p* < 0.001). Furthermore, male sex (OR = 0.35; 95% CI: 0.19–0.67; *p* < 0.002) and wild pig group size (OR = 0.90; 95% CI: 0.82–0.98; *p* = 0.016) were associated with decreased odds of PCV3 detection (Tables 5 and 6).

**Table 5:** Univariate mixed-effects logistic regression analysis of risk factors (categorical predictors) associated with PCV3-molecular detection in wild pigs captured in Alberta

Variable	Category	Positive (*n* = 90)	Negative (*n* = 241)	ß ^a^	SE ^b^	OR ^c^	95% CI ^d^	P-value
		N (%)	N (%)					
Sex (*n* = 331)	Female (*n* = 164)	52 (31.7)	112 (68.3)	-0.196	0.430	ref	**-**	**-**
	Male (*n* = 167)	38 (22.8)	129 (77.2)	-1.040	0.330	0.35	0.19–0.67	0.002*
Age (*n* = 331)	Juvenile (*n* = 121)	9 (7.4)	112 (92.6)	-4.168	0.727	ref	**-**	**-**
	Mature (*n* = 210)	81 (38.6)	129 (61.4)	3.955	0.699	52.2	13.3–205	< 0.001*
Pregnancy Status (*n* = 117) ^e^	No Pregnant (*n* = 87)	28 (32.2)	59 (67.8)	-1.194	0.678	ref	**-**	**-**
	Pregnant (*n* = 30)	13 (43.3)	17 (56.7)	0.680	0.744	1.97	0.46–8.48	0.361
County of captured (*n* = 331)	LSA (*n* = 77)	25 (32.5)	52 (67.5)	-0.902	0.835	ref	**-**	**-**
	WL (*n* = 222)	58 (26.1)	164 (73.9)	0.371	0.950	1.45	0.23–9.33	0.696
	TH (*n* = 21)	6 (28.6)	15 (71.4)	0.004	1.490	1.00	0.05–18.6	0.998
	SC (*n* = 11)	1 (9.1)	10 (90.9)	-2.550	2.039	0.08	0.00–4.25	0.211
Season of collection (*n* = 331)	Fall (*n* = 33)	9 (27.3)	24 (72.7)	0.312	0.292	ref	**-**	**-**
	Winter (*n* = 149)	59 (39.6)	90 (60.4)	2.400	2.480	2.40	0.32–18.1	0.395
	Spring (*n* = 116)	16 (13.8)	100 (86.2)	0.548	0.622	0.55	0.06–5.06	0596
	Summer (*n* = 33)	6 (18.2)	27 (81.8)	1.510	1.255	1.51	0.30–7.63	0.615
Period (*n* = 331)	2018–2021 (*n* = 164)	29 (17.7)	135 (82.3)	0.278	0.145	ref	**-**	**-**
	2022–2024 (*n* = 167)	61 (36.5)	106 (63.5)	2.370	1.610	2.37	0.63–8.98	0.202

^a^ ß: Estimate

^b^ SE: Standard Error

^c^ OR: Odds Ratio

^d^ CI: Confidence Interval

^e^ Data was not available for all individuals

* Statistical Significance

**Table 6:** Univariate mixed-effects logistic regression analysis of risk factors (continuous predictors) associated with PCV3-molecular detection in wild pigs captured in Alberta

Variable	ß ^a^	SE ^b^	OR ^c^	95% CI ^d^	*P*-value
Weight ^e^ (*n* = 330)	0.060	0.009	1.05	1.04–1.08	< 0.001*
Wild pigs group size (*n* = 331)	-0.188	0.176	0.90	0.82–0.98	0.016*
Commercial pig density (head/km^2^)	0.136	0.562	1.15	0.54–2.44	0.725
Small-scale pig density (head/km^2^)	-0.314	0.347	0.73	0.37–1.44	0.367
Distance to commercial pig farms (km)	-0.044	0.047	0.96	0.87–1.05	0.346
Distance to small-scale pig farms (km)	0.096	0.187	1.10	0.76–1.59	0.607
Distance to wild pig farms (km)	0.864	0.277	0.86	0.46–1.62	0.650

^a^ ß: Estimate

^b^ SE: Standard Error

^c^ OR: Odds Ratio

^d^ CI: Confidence Interval

^e^ Data was not available for all individuals

* Statistical Significance

Likewise, in the analysis comparing RVA-positive (*n* = 21) and RVA-negative (*n* = 71) wild pigs group size was associated with decreased odds of RVA detection (OR = 0.91; 95% CI: 0.84–1.00; *p* = 0.043, Supplementary Table S13). For *Metastrongylus* spp., comparison between positive (*n* = 76) and negative (*n* = 116) wild pigs showed that closer proximity to wild pig farms was significantly associated with parasite detection (OR = 0.85; 95% CI: 0.75–0.97; *p* = 0.017; Supplementary Table S16).

Risk factor analyses were also performed for PPV and *Eimeria* spp.; however, no significant associations were detected between the categorical or continuous predictor variables and pathogen detection (Supplementary Materials Table S14-S15).

## Discussion

Our findings indicate that free-ranging invasive wild pigs in Alberta can harbour multiple pathogens of both agricultural and zoonotic importance. Among the 17 pathogens investigated, evidence of prior exposure or infection was detected for 12 pathogens, including viral (PCVs, PRRSV, RVs, and PPV), bacterial (*E. rhusiopathiae*,* Mhyo*, and *Salmonella* spp.), and parasitic (*Eimeria* spp., *Metastrongylus* spp., *Trichuris* spp., *Ascaris* spp., and *Strongyloides* spp.).

Notably, antibody testing showed widespread exposure to *E. rhusiopathiae* in all wild pigs examined (100% overall). Although recent peer-reviewed publications documenting porcine erysipelas in Alberta are limited, the disease still occurs sporadically in domestic herds. Historical investigations have reported the occurrence in Canadian swine herds [17, 33]. High seroprevalence has also been reported in wild pig populations elsewhere, including 69.4% in Italy [34], 75.1% in Poland [35], and 95.6% in Japan [36]. The high seropositivity observed in Alberta suggests widespread and sustained exposure to *E. rhusiopathiae* within free-ranging wild pig population, indicating that transmission is likely well established in this population. Given that *E. rhusiopathiae* is a recognized zoonotic pathogen, these findings have limited but relevant public health implications. Human infections with *Erysipeloid* are uncommon and typically occur in individuals with occupational or recreational exposure to animals or carcasses [37]. While the absolute risk is low, documenting the presence of *E. rhusiopathiae* in wildlife populations helps inform occupational risk assessments and biosafety practices for those who routinely handle wild pigs.

PCVs were among the most frequently detected pathogens in this study. Molecular testing revealed a similar frequency of occurrence for PCV2 (32.9%) and PCV3 (27.2%). In contrast, PCV1 was detected only rarely (0.3%), consistent with its classification as a non-pathogenic circovirus with limited circulation in swine worldwide. PCV2 and PCV3 are recognized among the most economically significant viral pathogens affecting the global swine industry [38]. The high frequency of PCV2/PCV3 in Alberta wild pigs is consistent with the epidemiological patterns described in Canadian domestic pigs. Specifically, PCV2 has long been considered endemic in Alberta domestic swine herds [39], whereas PCV3 has been increasingly reported in Canadian pig populations, including Alberta [40].

Similarly, PCVs proportions detection rates reported in wild pig populations from other regions are comparable to those observed in the present study, including PCV2 and PCV3 rates of 57.7% and 15.4% in Brazil [41], and 76.7% and 55% in Portugal [42], and 58.3% and 10.9% in China [43]. Collectively, these findings suggest widespread circulation of both viruses in free-ranging wild pig populations worldwide.

We observed an association between mature individuals and PCV2/PCV3 positivity, and a lower probability of PCV3 detection in males compared to females. Previous studies have found a higher prevalence of PCVs infection in adult wild pigs [44], particularly in adult females [45]. In contrast, a recent study from Poland reported an overall PCV3 prevalence of 54% in wild pigs. Interestingly, the authors reported a higher frequency of PCV3 detection in animals younger than 12 months, notably only a small number of juveniles were included in the study [46]. The observed age and sex effects may be driven by cumulative exposure over time, differences in social behaviour and contact structure between sexes and age groups, longer survival of infected animals, or temporary protection from maternally derived antibodies in juveniles.

Body weight was also positively associated with PCV2/PCV3 detection. However, this relationship should be interpreted cautiously, as weight is strongly age-dependent, and only univariate analysis could be performed. Consequently, it was not possible to determine whether body weight represents an independent risk factor or whether the observed association simply reflects age-related differences. Furthermore, the broad ‘mature’ category was based on external morphological characteristic and therefore may have include individuals of multiple age classes.

We identified a significant co-detection between PCV2 and PCV3 (*p* < 0.001). Reports from other regions similarly describe frequent PCV2/PCV3 co-detection in free-ranging wild pigs, with documented proportions ranging from approximately 7.5 and 23% in Europe and Asia [47, 48]. This co-detection has been attributed to shared transmission pathways, overlapping ecological niches, and potential synergistic interactions between circoviruses [49], which may facilitate concurrent circulation within wild pig populations.

In addition, we found common gastrointestinal and respiratory parasites in the Alberta wild pig population, with *Eimeria* spp. (39.6%) and *Metastrongylus* spp. (25.5%) being the most predominant. Species-level identification was not possible for most parasite groups because fecal flotation detects morphologically similar eggs that cannot be reliably differentiated without sporulation, larval culture, or molecular methods. Parasite transmission in free-ranging pigs is strongly influenced by environmental contamination, high host density, and fecal–oral exposure, and the group-living behaviour observed in our study likely facilitates spread [50].

*Metastrongylus* spp. infections, which depend on earthworm intermediate hosts, are uncommon in indoor domestic swine but expected in outdoor or free‑ranging populations. Wild pigs in Alberta routinely root in soil‑rich habitats [2], and multiple *Metastrongylus* species have been reported in wild pigs elsewhere [51]. In the present study *Metastrongylus* spp. detection was also associated with closer proximity to wild pig farms. Although this study cannot determine whether this association reflects parasite transmission between captive and free-ranging wild pigs or shared environmental conditions that favor parasite persistence, it highlights a potential epidemiological link that warrants further investigation. Similarly, *Metastrongylus* spp. has been reported in farmed wild boars in Finland, highlighting its relevance in outdoor pig production systems [52].

Other gastrointestinal nematodes, including *Ascaris* spp. (5.7%), *Trichuris* spp. (5.7%), and *Strongyloides* spp. (4.2%), occurred at low levels. A Saskatchewan study similarly detected *Trichuris* spp. and *Metastrongylus* spp. but not *Ascaris* spp. or *Strongyloides* spp [28]. Importantly, *Ascaris suum*, *Trichuris suis*, and *Strongyloides ransomi* are recognized zoonotic nematodes [53].

Porcine rotaviruses (both RVA and RVC) are major causes of diarrhea in pigs [54], and are endemic in Canada [55]. Zoonotic spillover of RVC from pigs to humans appears to be a rare event, with phylogenetic evidence reported in several countries [24, 56]. In contrast, the zoonotic potential of RVA strains has been documented repeatedly [57].

Within Canada, surveillance of domestic swine herds in Ontario and Quebec reported high detection proportions of RVA (69.9%), RVB (32.6%), and RVC (53.1%) [15]. Compared with the high prevalence in Canadian domestic swine, Alberta wild pigs exhibited lower proportions of RVA (22.8%) and RVC occurrence (5.4%). Internationally, RVA infection has been previously described in wild pig populations, with reported prevalences of 1% in Germany [58], 4.4% in Japan [59], and 9.3% in Croatia [60]. RVC has been scarcely investigated in wild pigs, with a prevalence of 12.8% in the Czech Republic [61]. The occurrence of RVA and RVC in Alberta wild pigs falls within the range reported for free-ranging wild pig populations internationally.

RVA has a broad host range, including bats, rodents, shrews, carnivores, and wild ungulates [58], its detection in Alberta wild pigs suggests that this species could play a role in the ecology of rotaviruses at the wildlife–livestock interface. Direct or indirect contact between wild pigs, domestic livestock, and native wildlife could provide opportunities for virus transmission among susceptible host populations. Whether wild pigs facilitate virus exchange among these host populations remains unknown and warrants further investigation.

PPV is considered endemic in swine populations across Canada [13], however, contemporary epidemiological data remain limited. Most available information is derived from a historical serological survey conducted in Ontario in the late 1970s, reporting 76% seropositivity [62]. A more recent Canadian study focused on high-throughput sequencing, reporting a new PPV genotype [63].

In the present study, PPV was detected in 17.8% of tested wild pigs, indicating active virus circulation within - Alberta free-ranging populations. Similar evidence of PPV exposure has been reported in wild pigs internationally, although prevalence appears highly variable among regions, ranging from 4 to 10% in Turkey and Spain [64, 65] to substantially higher levels in Italy (44%), Germany (64.3%), and Sweden (78%) [66–68].

The detection of PPV in Alberta wild pigs raises the possibility that the virus may be maintained within free-ranging populations. Molecular studies from Romania have shown that PPV circulating in wild pigs is genetically more diverse than that found in domestic pigs and may include antigenically distinct variants [69]. Although wild pigs are not currently considered a major reservoir for PPV transmission to domestic swine [70], contact between wild and domestic pig populations could provide opportunities for virus transmission. Such transmission could facilitate the introduction of genetically distinct PPV variants into domestic swine populations, with potential implications for vaccine effectiveness [71] and the occurrence of PPV-associated reproductive failure, including SMEDI syndrome (Stillbirth, Mummification, Embryonic Death, and Infertility) [72].

*Salmonella* infections is one of the leading causes of bacterial enteritis in Canadian swine herds [23], and represents an important foodborne pathogen associated with pork production [73]. *Salmonella* spp. identified in our study were related to *S. enterica* serotype Montevideo (MV) and *S. enterica* subsp. *diarizonae* serotype 61: k:1,5, (7) (SED). MV is a well-recognized foodborne pathogen frequently implicated in human outbreaks and contamination of pork and other food products [74]. In contrast, SED is typically associated with sheep and ruminants [75, 76], as well as a wide range of wildlife species [77, 78]. Detection of SED in wild pigs is generally uncommon, with low prevalence reported mostly in Europe [79, 80]. SED has additionally been documented as an opportunistic zoonotic pathogen capable of causing human infections, particularly in immunocompromised individuals [81].

The detection of these serotypes in wild pigs suggests several plausible exposure routes, including occasional scavenging of wild carcasses, indirect contact with livestock, and potential environmental acquisition from soil or water, as *Salmonella* spp. is known to persist in these substrates [82]. Although the overall occurrence of *Salmonella* spp. was low (2.8%), the presence of zoonotic serotypes highlights the potential of free-ranging wild pigs to serve as incidental carriers and underlines the importance of monitoring *Salmonella* spp. at the wildlife–livestock–human interface.

PRRSV and *Mhyo* are economically important endemic pathogens of the Canadian swine industry [83, 84]. Previous studies in Canadian swine herds reported PRRSV seroprevalence of 38.3–65%, and *Mhyo* seroprevalence of 26.7–85% [12]. In contrast, serological evidence of exposure to both pathogens was limited in Alberta wild pigs, with PRRSV and *Mhyo* detected in 1% and 2.1%, respectively. Similar findings were reported in wild pigs from Saskatchewan, Canada, where no serological evidence of PRRSV or *Mhyo* exposure was detected [28].

The low occurrence of PRRSV and *Mhyo* observed in the present study is also consistent with previous investigations in wild pigs internationally. PRRSV exposure in wild pig populations has often been absent [28, 68, 85] or detected only at very low seroprevalence, typically ranging from 2.5% to 5.6% in the United States [27] and Europe [67]. Reports of *Mhyo* in wild pigs are more variable. Several studies have reported no evidence of infection [86, 87] or only sporadic detection [88, 89]. However, a recent molecular investigation documented a substantially higher prevalence (41.9%) in Polish wild pigs [90].

Interestingly, we identified a single individual seropositive for PRRSV type 1 (European genotype). Although PRRSV type 2 predominates in North America, occasional detections of PRRSV type 1 have also been reported in the United States (2.15%) [91]. Nevertheless, this finding should be interpreted carefully because the ELISA S/P value (0.36) was close to manufacturer’s positivity threshold (0.30), the animal was RT-qPCR negative, no additional serological confirmation was performed, and potential cross-reactivity between PRRSV type 1 (EU) and type 2 (NA) antibodies has been reported for this assay.

Together, these results indicate that the low occurrence of PRRSV and *Mhyo* reflects only sporadic exposure within Alberta free-ranging wild pigs, suggesting that these individuals are unlikely to represent significant reservoirs for either pathogen.

Overall, these findings have epidemiological implications for domestic swine health in Alberta. Wild pigs are attracted to agricultural lands by the availability of food resources, increasing the likelihood of contact with domestic pigs [4]. This risk is particularly relevant for small-scale production systems, where biosecurity practices are limited [92]. Although our data cannot demonstrate bidirectional transmission, the detection of pathogens frequently reported in commercial swine herds raises questions regarding the potential sharing of pathogens between wild and domestic pigs. Given that Alberta wild pig populations originated from escaped farmed wild boars, one possible explanation is that the pathogens detected in this study may reflect historical introductions resulting from infections acquired during the farming period. However, continued detection of these pathogens years after population establishment, including in individuals classified as juveniles, which represent wild pigs born within the free-ranging population, supports the hypothesis of pathogen maintenance through ongoing transmission and circulation within the free-ranging wild pig populations. Alternatively, or additionally, pathogen exposure may result from spillover through direct or indirect contact with domestic pigs. Together, these findings highlight the need to understand the direction, frequency, and mechanisms of pathogen exchange at this wildlife–livestock interface, including ongoing spillover from domestic pigs, maintenance within wild pig populations, and potential spillback into commercial herds.

Future research integrating genomic comparison such as network analysis, phylogenetic studies, or whole-genome sequencing are needed to compare circulating strains between host populations and provide stronger evidence for potential transmission pathways. Expanding surveillance to include additional production-limiting pathogens, such as *Actinobacillus pleuropneumoniae*, together with broader geographic sampling, inclusion of carcass-based surveillance, and diagnostic testing across all collected individuals, would improve the accuracy of pathogen occurrence and strengthen the detection of emerging threats.

A limitation in this study is that wild pigs were captured through the Alberta Wild Boar Control Program, which uses whole-sounder trapping in areas with confirmed sightings or high expected wild pig density. Consequently, sampling was geographically clustered, primarily within central Alberta, reflecting where most established wild pig populations currently occur. This live-capture method also inherently favours healthy, mobile individuals, whereas sick, debilitated, or moribund wild pigs are less likely to approach baited traps and were therefore probably underrepresented. No wild pig carcasses associated with mortality events were found or sampled during the study period, limiting the ability to detect outbreaks or spillover events.

Serum availability further constrained interpretation. Blood samples were obtained during field sampling, and logistic limitations resulted in serum collection for only 120 of the 331 wild pigs, all from 2021 to 2024. This restricted temporal coverage may limit the interpretation of exposure trends across the full study period.

Similarly, the absence of IAV, SVA, PDCoV, PEDV, and TGEV detections should also be interpreted cautiously. PCR‑based surveillance reflects only active shedding at the time of sampling and may underestimate true exposure, particularly for pathogens with short shedding windows or seasonal transmission patterns.

The pathogen co-detection estimates in this study are constrained by differences in diagnostic methods and unequal testing coverage across individuals. Only molecular and coprological results were used, and several pathogens were excluded because diagnostic outcomes were incomplete. As a result, co-detection frequencies were calculated from a restricted subset of animals which may not fully represent the broader wild pig population.

## Conclusions

In summary, this study provides the first multi-pathogen assessment of free-ranging invasive wild pigs in Alberta and shows that these populations carry several agents of relevance for swine health, wildlife management, and public health. Of the examined pathogens, viral, bacterial, and parasitic were detected, including *E. rhusiopathiae*, PCVs, nematodes and coccidia, RVs, *Salmonella* spp., PRRSV, *Mhyo* and PPV. Although not all screened pathogens were detected, the presence of swine production-limiting and zoonotic agents indicates that wild pigs may contribute to the broader epidemiological landscape of diseases affecting domestic livestock and humans. Collectively, these results highlight the value of wildlife disease surveillance, as monitoring pathogen circulation in wild pigs supports early detection of emerging threats and strengthens risk assessments for domestic swine and human health.

## Supplementary Information

Supplementary Material 1. S1- Bacteriological Testing Procedures for *Salmonella spp*. Supplementary Table S1–Overview of nucleic acid extraction methods used for target pathogens before amplification. Supplementary Table S2–Real-time PCR for the detection of *Salmonella spp*. Supplementary Table S2.1–Thermocycler conditions for the detection of *Salmonella spp*. Supplementary Table S3–Real-time reverse transcription-PCR (RRT-PCR) protocol and sequencing primers-probes used for the detection of Seneca virus A (SVA). Supplementary Table S3.1–Thermocycler conditions for the detection of SVA. Supplementary Table S4–Real-time reverse transcription-PCR (RRT-PCR) for the detection of porcine epidemic diarrhea virus (PEDV), porcine deltacoronavirus (PDCoV), and transmissible gastroenteritis virus (TGEV). Supplementary Table S4.1–Thermocycler conditions for the detection of PEDV, PDCoV, and TGEV. Supplementary Table S5–Real-time reverse transcription-PCR (RRT-PCR) for the detection of influenza A virus (IAV). Supplementary Table S5.1–Thermocycler conditions for the detection of IAV. Supplementary Table S6–Real-time reverse transcription-PCR (RRT-PCR) protocol and sequencing primers-probes used for the detection of porcine circovirus (PCVs). Supplementary Table S6.1–Thermocycler conditions for the detection of PCVs. Supplementary Table S7–Real-time reverse transcription-PCR (RRT-PCR) protocol and sequencing primers-probes used for the detection of porcine parvovirus (PPV). Supplementary Table S7.1–Thermocycler conditions for the detection of PPV. Supplementary Table S8–Thermocycler conditions for the detection of rotavirus (RVs) subtypes RVA, RVB, and RVC. Supplementary Table S9–Thermocycler conditions for the detection of porcine reproductive and respiratory syndrome virus (PRRSV). Supplementary Table S10–Summary of Alberta wild pigs tested for target pathogens across sampling years (2018–2024). Supplementary Table S11–Post hoc minimum detectable prevalence estimates for pathogens tested. Supplementary Table S12–Description and calculation of spatial covariates included in the mixed-effects logistic regression models. Supplementary Table S13–Univariate mixed-effects logistic regression analysis of risk factors (categorical predictors) associated with RVA-molecular detection in wild pigs captured in Alberta. Supplementary Table 13.1–Univariate mixed-effects logistic regression analysis of risk factors (continuous predictors) associated with RVA-molecular detection in wild pigs captured in Alberta. Supplementary Table 14–Univariate mixed-effects logistic regression analysis of risk factors (categorical predictors) associated with PPV-molecular detection in wild pigs captured in Alberta. Supplementary Table 14.1–Univariate mixed-effects logistic regression analysis of risk factors (continuous predictors) associated with PPV-molecular detection in wild pigs captured in Alberta. Supplementary Table 15–Univariate mixed-effects logistic regression analysis of risk factors (categorical predictors) associated with Eimeria spp.-coprological detection in wild pigs captured in Alberta. Supplementary Table 15.1–Univariate mixed-effects logistic regression analysis of risk factors (continuous predictors) associated with Eimeria spp.-coprological detection in wild pigs captured in Alberta. Supplementary Table 16–Univariate mixed-effects logistic regression analysis of risk factors (categorical predictors) associated with *Metastrongylus spp.*-coprological detection in wild pigs captured in Alberta. Supplementary Table 16.1–Univariate mixed-effects logistic regression analysis of risk factors (continuous predictors) associated with *Metastrongylus spp.*-coprological detection in wild pigs captured in Alberta. Supplementary Fig. S1–Parasite species identified in Alberta wild pigs from 2018-2024. (A, B) *Eimeria spp. oocysts*; (C, D) *Metastrongylus spp.* eggs; (E, F) *Trichuris* spp. eggs; and (G, H) Ascaris spp. eggs. Supplementary Statistical Analysis Methods References

Supplementary Material 2. R scripts- Statistical Analysis.

## Data Availability

The data generated or analyzed during this study are owned by the Government of Alberta. Due to access restrictions, these data are not publicly available. They can be made available upon reasonable request by contacting the corresponding author and with authorization from the Government of Alberta.

## Abbreviations

PRRSV: Porcine reproductive and respiratory syndrome virus
PRRSV types 1 (EU): Porcine reproductive and respiratory syndrome virus genotype/type 1 European Union
PRRSV genotype/type 2 (NA): Porcine reproductive and respiratory syndrome virus types 2 North America
PCVs: Porcine circoviruses
PCV1: Porcine circovirus type 1
PCV2: Porcine circovirus type 2
PCV3: Porcine circovirus type 3
Mhyo: Mycoplasma hyopneumoniae
PPV: Porcine parvovirus
IAV: Influenza A virus
RVs: Rotaviruses
RVA: Rotavirus A
RVB: Rotavirus B
RVC: Rotavirus C
E. rhusiopathiae: Erysipelothrix rhusiopathiae
PDCoV: Porcine deltacoronavirus
PEDV: Porcine epidemic diarrhea virus
TGEV: Transmissible gastroenteritis virus
SVA: Seneca virus A
FMD: Foot-and-mouth disease
SED: Salmonella enterica subsp. diarizonae serotype 61:k:1,5, (7)
MV: Salmonella enterica serotype Montevideo
EIM: Eimeria spp
MET: Metastrongylus spp
TRI: Trichuris spp
ASC: Ascaris spp
STR: Strongyloides spp
BCS: Body condition score
WL: Woodland county
LSA: Lac Ste. Anne county
TH: Two Hills county
SC: Strathcona county
kg: Kilograms
AGI: Animal Health Section, Animal Health and Assurance Branch, Primary Agriculture Division, Alberta Agriculture and Irrigation
DSU: Diagnostic Services Unit
WOAH: World Organization for Animal Health
ELISA: Enzyme-linked immunosorbent assay
S/P: Sample/Positive or Sample-to-Positive ratio
PCR: Polymerase Chain Reaction
CI: Confidence intervals
p: p-value
ϕ: Phi Coefficient
FDR: False discovery rate
AIC: Akaike’s Information Criteria
AUC-ROC: Area Under the Receiver Operating Characteristic Curve
SD: Standard Deviation
ß: Estimate
SE: Standard Error
OR: Odds Ratio
n: Total of samples tested
AVMA: American Veterinary Medical Association, and OCPV: Office of the Chief Provincial Veterinarian
