# Supplementary material for: Surveillance of zoonotic and production-limiting pathogens in invasive free-ranging wild pigs in Alberta, Canada
Source: BMC Vet Res. 2026 Aug 5;22:654. doi: 10.1186/s12917-026-05793-1 (PMC13729470; doi:10.1186/s12917-026-05793-1)
Supplement: Supplementary file 1 [file 12917_2026_5793_MOESM1_ESM.docx]

**Additional File**

**For**

**Surveillance of zoonotic and production-limiting pathogens in invasive free-ranging wild pigs in Alberta, Canada**

Oshin Ley-Garcia^1*^, Chunu Mainali^2^, Frank van der Meer^1^, Davor Ojkic^3^, Carolina Malgarin^4^, Bryna Turk^1^, Sylvia L. Checkley^1^, Hannah McKenzie^2^, and Mathieu Pruvot^1,5^

***** Corresponding author

**Supplementary Material 1: S1-Bacteriological Testing Procedures for *Salmonella* spp.**

Wild pig fecal samples (approximately 2 g), collected from the intestine during necropsy, were diluted in 18 mL of Buffered Peptone Water (BPW) under aseptic conditions and incubated at 35°C for 24 h for pre-enrichment. From the pre‑enriched BPW sample, aliquots were inoculated into two selective enrichment broths in parallel. Specifically, 1 mL of the pre‑enriched BPW was transferred into 10 mL of Tetrathionate (TT) Broth (Difco, BD Diagnostics, Sparks, MD, USA) supplemented with 0.2 mL of iodine. Then, 0.1 mL of the pre‑enriched BPW was transferred into 9.9 mL of Rappaport‑Vassiliadis Soya (RV) Broth (MilliporeSigma, Burlington, MA, USA). Both TT and RV broths were incubated at 35°C for 24 h.

After incubation, 500 μl of each enrichment broth was combined for DNA extraction using the MagneSil KF Genomic System Kit (Promega, USA), following the manufacturer’s instructions. The resulting DNA was analyzed by RT-PCR to confirm the presence of *Salmonella* spp. (Supplementary Information, Tab.S1).

Additionally, 100 μl of TT Broth was transferred onto Modified Semi‑Solid Rappaport‑Vassiliadis (MSRV) plates (Sigma-Aldrich, Oakville, ON, CA) and incubated overnight at 42°C. For samples testing positive by RT-PCR, 10 μl aliquots of the TT and RV broths were transferred onto selective agar. TT broth was plated onto XLT4 agar (Sigma-Aldrich, Oakville, ON, CA) and Rambach (RAM) agar (Chromocult Sigma-Aldrich, Oakville, ON, CA), while RV was plated onto XLT4, RAM and BG Sulfa (BGS) agar (Difco, BD Diagnostics, Sparks, MD, USA). MSRV plates were examined after 20 h, and growth from characteristic motility “halos’’ was subcultured onto RAM and XLT4 agar. All selective agar plates were incubated at 35°C for 24 h.

Presumptive *Salmonella* spp. colonies from selective agar were biochemically screened using Triple Sugar Iron (TSI) agar (Difco, BD Diagnostics, Sparks, MD, USA), Lysine Iron Agar (LIA; Oxoid, Basingstoke, Hampshire, UK), and Urea (URE) agar (BBL, BD Diagnostics, Sparks, MD, USA), and incubated at 35°C for 24 h to assess characteristic biochemical reactions. Colonies with typical biochemical reactions were streaked onto MacConkey (MAC) agar (BBL) and Columbia Blood Agar (Difco, BD Diagnostics, Sparks, MD, USA) and incubated at 35°C for 24 h to confirm purity.

Biochemically typical isolates of *Salmonella* spp. were tested serologically using the *Salmonella* Latex Agglutination Test Kit (Oxoid, Basingstoke, Hampshire, UK) and *Salmonella* Poly O, O1, O9‑O antisera (Denka Seiken, Tokyo, Japan). Isolates showing typical biochemical profiles and positive agglutination were considered positive for *Salmonella* spp. *Salmonella enterica* subsp. *enterica* *serovar* *Typhimurium* ATCC 14028 was used as a positive control.

**Supplementary Material 2: Supplementary Table S1-**Overview of nucleic acid extraction methods used for target pathogens before amplification.

| **Extraction Kit** | **Nucleic acid extraction** | **Supplier** | **Target Pathogen^a^** | **Sample Used** |
| --- | --- | --- | --- | --- |
| MagMAX Viral RNA Isolation Kit | Total nucleic acids | ThermoFisher Scientific, Waltham, MA, USA | SVA | Feces and Nasal Swabs |
|  |  |  | PEDV | Feces |
|  |  |  | PDCov | Feces |
|  |  |  | TGEV | Feces |
|  |  |  | IAV | Nasal Swabs |
|  |  |  | RVA, RVB, RVC | Feces |
|  |  |  | PRRS | Spleen |
|  | DNA |  | PCV1, PCV2, PCV3 | Spleen |
|  |  |  | PPV | Spleen |
| MagneSil KF Genomic System Kit |  | Promega, USA | *Salmonella* spp. | Bacterial enrichment cultures |
| Qiagen DNeasy Blood & Tissue Kit |  | Qiagen, Toronto, Ontario, CA | *Mycoplasma* spp. | Lungs |

**^a^**SVA: Seneca virus A, PEDV: porcine epidemic diarrhea virus, PDCoV: porcine deltacoronavirus, TGEV: transmissible gastroenteritis virus, IAV: Influenza A virus, RV: Rotavirus, PRRS: Porcine reproductive and respiratory syndrome, PCV: Porcine circovirus, and PPV: Porcine parvovirus.

**Supplementary Material 3: Supplementary Table S2–**Real-time PCR for the detection of *Salmonella* spp.

| **Specificity** | **Gene** | **Primer-Probe Name** | **Sequence (5’-3’)** | **Amplicon size (bp)** | **Orientation** | **Final**  **conc.** | **Reference** |
| --- | --- | --- | --- | --- | --- | --- | --- |
| *Salmonella* spp. | invA | LC-Sal1 | AAC TTC ATC GCA CCG TCA | 219 | Forward | 400 nM | [1] |
|  |  | LC-Sal2 | ATT GTC ACC GTG GTC CAG |  | Reverse |  |  |
|  |  | LC-Sal1 Probe 1 | CGC ATC AAT AAT ACC GGC CTT CA-FL |  |  | 200 nM |  |
|  |  | LC-Sal1 Probe 2 | LC610-TCGGCATCAATACTCATCTGTTTACCG |  |  |  |  |
|  |  | LC-Sal IC Probe 1 | CGT TGG CTA CCC GTG ATA TTG C-FL |  |  |  |  |
|  |  | LC-Sal IC Probe 2 | LC670-GAA GAG CTT GGC GGC GAAT |  |  |  |  |

**Supplementary Material 3: Supplementary Table S2.1–**Thermocycler conditions for the detection of *Salmonella* spp.

| **Steps** | **Temperature** | **Time** | **Cycle** |
| --- | --- | --- | --- |
| Initial denaturation | 95°C | 60 s | 1 |
| Denaturation | 95°C | 15 s | 40 |
| Annealing/Extension | 60°C | 30 s |  |
| Hold | 4°C | ∞ | **-** |

The reactions were prepared in 20 μL, containing 10 uL of 1X Luna Universal Probe qPCR Master Mix (New England Biolabs), and 2 uL of DNA template. PCR reactions were all set in 96-well optical plates (Applied Biosystems, Thermo Fisher Scientific).

A genomic DNA control of *Salmonella enterica* subsp. *enterica* serovar Typhimurium (ATCC 700720D-5; Cedarlane Labs, Mississauga, ON, Canada) was included as a positive control. Samples with a cycle threshold (*C*_t_) value below 30 were interpreted as positive.

**Supplementary Material 4: Supplementary Table S3–**Real-time reverse transcription-PCR (RT-qPCR) protocol and sequencing primers-probes used for the detection of Seneca virus A (SVA).

| **Specificity** | **Gene** | **Primer-Probe Name** | **Sequence (5’-3’)** | **Amplicon size (bp)** | **Orientation** | **Final**  **conc.** | **Reference** |
| --- | --- | --- | --- | --- | --- | --- | --- |
| SVA | SVA 2C | SVA 4269-For | TCT CTT GCC CTA ACA CTG GGG | 117 | Forward | 200 nM | [2] |
|  |  | SVA 4385-Rev | CTT GCC TCT AAG GAC CAC CACA |  | Reverse |  |  |
|  |  | SVA FAM Probe | 6FAM- TGG CCC AAA/ZEN†/GTC TCA CCA CTA TGA TCA ATG -IABkFQ† |  |  | 100 nM |  |

†ZEN/3IABkFQprobe IDT specific

**Supplementary Material 4: Supplementary Table S3.1–**Thermocycler conditions for the detection of SVA.

| **Steps** | **Temperature** | **Time** | **Cycle** |
| --- | --- | --- | --- |
| Reverse transcription | 48°C | 10 min | 1 |
| Initial denaturation | 95°C | 10 min | 1 |
| Denaturation | 95°C | 10 s | 40 |
| Annealing/Extension | 60°C | 60 s |  |
| Hold | 4°C | ∞ | **-** |

RT-qPCR was performed in a total volume of 25 μL containing 2X RT-PCR Buffer of AgPath-ID™ Mix (Applied Biosystems, Waltham MA, USA), 25X RT-PCR Enzyme Mix, and 5 uL of RNA template. Reactions were prepared in 96-well optical plates (Applied Biosystems, Thermo Fisher Scientific).

Each run included a negative control (RNase-free water) and a positive control (SVA gBlock RNA control synthesized as a Gene Fragment, IDT Technologies, Coralville, Iowa, USA).

Samples with a cycle threshold (*C*_t_) value below 35.99 were considered positive.

**Supplementary Material 5: Supplementary Table S4–**Real-time reverse transcription-PCR (RT-qPCR) for the detection of porcine epidemic diarrhea virus (PEDV), porcine deltacoronavirus (PDCoV), and transmissible gastroenteritis virus (TGEV).

| **Specificity** | **Gene** | **Primer-Probe Name** | **Sequence (5’-3’)** | **Amplicon size (bp)** | **Orientation** | **Final**  **conc.** | **Reference** |
| --- | --- | --- | --- | --- | --- | --- | --- |
| PEDV | PEDV N | PEDV- F | TAT GCT CAG ATC GCC AGT | 63 | Forward | 400 nM | [3] |
|  |  | PEDV- R | AGC CAC ATT ACC ACC AAAG |  | Reverse |  |  |
|  |  | PEDV FAM Probe | FAM-AGCAATGCTGCAACATTTGGTGC-BHQ1 |  |  | 120 nM |  |
| PDCoV | PDCoV N | PDCoV - F | AGC CAG AGA GCC AGT CA | 146 | Forward | 400 nM |  |
|  |  | PDCoV - R | TGT ACC CTC GAT CGT ACT CC |  | Reverse |  |  |
|  |  | PDCoV HEX Probe | HEX-ATGCAAACTAGGGCTGGCTACTCT-BHQ1 |  |  | 120 nM |  |
| TGEV | TGEV N | TGEV - F | TCT GCT GAA GGT GCT ATT ATA TGC | 146 | Forward | 400 nM |  |
|  |  | TGEV - R | CCA CAA TTT GCC TCT GAA TTA GAAG |  | Reverse |  |  |
|  |  | TGEV HEX Probe | HEX-YAAGGGCTCACCACCTACTAC-BHQ1 |  |  | 120 nM |  |

**Supplementary Material 5: Supplementary Table S4.1–**Thermocycler conditions for the detection of PEDV, PDCoV, and TGEV.

| **Steps** | **Temperature** | **Time** | **Cycle** |
| --- | --- | --- | --- |
| Reverse transcription | 45°C | 10 min | 1 |
| Initial denaturation | 95°C | 15 min | 1 |
| Denaturation | 95°C | 15 s | 40 |
| Annealing/Extension | 60°C | 60 s |  |
| Hold | 4°C | ∞ | **-** |

RT-qPCR assays were performed in a total reaction volume of 25 µL, consisting of 12.5 µL of 2X AgPath‑ID™ RT‑PCR Buffer (Applied Biosystems, Waltham, MA, USA), 1 µL of 25X RT‑PCR Enzyme Mix, and 5 µL of extracted genomic RNA template. The reactions were mixed in 96-well optical plates (Applied Biosystems, Thermo Fisher Scientific).

For each batch of samples, a negative control (RNase-free water) and positive controls-PEDV and PDCoV (gBlock RNA control synthesized as a Gene Fragment; IDT Technologies, Coralville, Iowa, USA) and TGEV (ATCC VR-174) were included. Samples with a *C*_t_<37 were considered positive.

**Supplementary Material 6: Supplementary Table S5–**Real-time reverse transcription-PCR (RT-qPCR) for the detection of influenza A virus (IAV).

| **Specificity** | **Gene** | **Primer-Probe Name** | **Sequence (5’-3’)** | **Amplicon size (bp)** | **Orientation** | **Final**  **conc.** | **Reference** |
| --- | --- | --- | --- | --- | --- | --- | --- |
| Influenza A  2009 Matrix | Matrix | Inf-A 2009  matrix- For | AGA TGA GTC **Y^1^**TC TAA CCG AGG TCG | 99 | Forward | 400 nM | [4] |
|  |  | Inf-A 2009  matrix- Rev | TGC AAA **R^2^**AC A**Y^1^Y^1^** TTC **M^3^**AG TCT CTG |  | Reverse |  |  |
|  |  | Inf-A matrix  M +64 probe | FAM-TCA GGC CCC CTC AAA GCC GA-BHQ1^6^ |  |  | 120 nM |  |

^1^**Y** = C or T; ^2^**R** = G or A ^3^**M** = A or C ^4^**W** = A or T

**Supplementary Material 6: Supplementary Table S5.1–**Thermocycler conditions for the detection of IAV.

| **Steps** | **Temperature** | **Time** | **Cycle** |
| --- | --- | --- | --- |
| Reverse transcription | 45°C | 10 min | 1 |
| Initial denaturation | 95°C | 10 min | 1 |
| Denaturation | 94°C | 5 s | 40 |
| Annealing/Extension | 60°C | 1 min |  |
| Hold | 4°C | ∞ | **-** |

The assay was performed using 25 μL reaction mixtures containing 12.5 uL of 2X RT-PCR Buffer of AgPath-ID™ Mix (Applied Biosystems, Waltham MA, USA), 1 uL of 25 X RT-PCR Enzyme Mix, and 4 μL of [template](https://www-sciencedirect-com.ezproxy.lib.ucalgary.ca/topics/biochemistry-genetics-and-molecular-biology/dna-template) RNA. The RT-qPCR reactions were set in 96-well optical plates (Applied Biosystems, Thermo Fisher Scientific).

*In vitro* transcribed (IVT) RNA controls for the Matrix (M) gene, prepared and supplied by CAHSN-NCFAD, were used as positive controls. Negative controls (RNase-free water) and positive RNA controls were included in each run. Samples yielding a cycle threshold (*C*_t_) value below 35.99 were interpreted as positive.

**Supplementary Material 7: Supplementary Table S6–**Real-time reverse transcription-PCR (RT-qPCR) protocol and sequencing primers-probes used for the detection of porcine circovirus (PCVs).

| **Specificity** | **Gene** | **Primer-Probe Name** | **Sequence (5’-3’)** | **Amplicon size (bp)** | **Orientation** | **Final**  **conc.** | **Reference** |
| --- | --- | --- | --- | --- | --- | --- | --- |
| PCV1 | Capsid | PCV1_Cap-For | CTC CGG AGA AGA CCA TAT TT | 76 | Forward | 500 nM | Designed internally ^a^ |
|  |  | PCV1_Cap-Rev | TGA AGA TAC CCG TCT TTC G |  | Reverse |  |  |
|  |  | PCV1_Pr | FAM-AAA CCG TTA CAG ATG GCG C |  |  | 250 nM |  |
| PCV2 | Rep | PCV2_Rep-For | TTG GAA GAA TGC TAC AGA ACA A | 78 | Forward | 500 nM | Designed internally ^a^ |
|  |  | PCV2_Rep-Rev | GGA AAT TCA GGG CAT GGG |  | Reverse |  |  |
|  |  | PCV2_Pr | HEX-AAA GGG TGA CGA ACT GGC CC |  |  | 250 nM |  |
| PCV3 | Capsid | PCV3_Cap-For | AGT GCT CCC CAT TGA ACG | 135 | Forward | 500 nM | [5] |
|  |  | PCV3_Cap-Rev | ACA CAG CCG TTA CTT CAC |  | Reverse |  |  |
|  |  | PCV3_Pr | TexRd-ACC CCA TGG CTC AAC ACA TAT GAC C |  |  | 250 nM |  |
| Enterovirus | Enterovirus 5' UTR  Internal control | Entero_31-For | ATG CGG CTA ATC CCA ACC T | 61 | Forward | 500 nM | CFIA ^b^ |
|  |  | Entero_31-Rev | CGT TAC GAC AGG CCA ATC ACT |  | Reverse |  |  |
|  |  | Entero_LNA-Pr | Cy5-CA+G+GTGGTCA+C+AAAC |  |  | 250 nM |  |

^A^ Primer-Probe were designed internally in the accredited Animal Health Laboratory (ISO17025) at the University of Guelph, Guelph, ON, Canada

^b^ Centre for Foreign Animal Disease (NCFAD), Canadian Food Inspection Agency (CFIA), Winnipeg, MB, R3E 3M4, Canada.

**Supplementary Material 7: Supplementary Table S6.1–**Thermocycler conditions for the detection of PCVs.

| **Steps** | **Temperature** | **Time** | **Cycle** |
| --- | --- | --- | --- |
| Reverse transcription | 45°C | 10 min | 1 |
| Initial denaturation | 95°C | 10 min | 1 |
| Denaturation | 94°C | 5 s | 45 |
| Annealing/Extension | 60°C | 1 min |  |
| Hold | 4°C | ∞ | **-** |

A multiplex RT-qPCR was performed using the TaqMan™ Fast Virus 1-Step Master Mix (ThermoFisher), in a total reaction volume of 20 µL containing 8 µL of template.

Samples with a Ct <37 were considered positive, and samples with a Ct ≥37 were considered inconclusive. If no amplification was detected, the sample was considered negative.

**Supplementary Material 8: Supplementary Table S7–**Real-time reverse transcription-PCR (RT-qPCR) protocol and sequencing primers-probes used for the detection of porcine parvovirus (PPV).

| **Specificity** | **Gene** | **Primer-Probe Name** | **Sequence (5’-3’)** | **Amplicon size (bp)** | **Orientation** | **Final**  **conc.** | **Reference** |
| --- | --- | --- | --- | --- | --- | --- | --- |
| PPV | VP2 coding region | PPV-PFw | CAG AAT CAG CAA CCT CAC CA | 106 | Forward | 500 nM | [6] |
|  |  | PPV-PRw | GCT GCT GGT GTG TAT GGA AG |  | Reverse |  |  |
|  |  | PPV-Pr | FAM-TGC AAG CTT AAT GGT CGC ACT AGA CA-BHQ |  |  | 250 nM |  |
| Enterovirus | Enterovirus 5' UTR  Internal control | Entero_31-For | ATG CGG CTA ATC CCA ACC T | 61 | Forward | 500 nM | CFIA ^a^ |
|  |  | Entero_31-Rev | CGT TAC GAC AGG CCA ATC ACT |  | Reverse |  |  |
|  |  | Entero_LNA-Pr | Cy5-CA+G+GTGGTCA+C+AAAC |  |  | 250 nM |  |

^a^ Centre for Foreign Animal Disease (NCFAD), Canadian Food Inspection Agency (CFIA), Winnipeg, MB, R3E 3M4, Canada.

**Supplementary Material 8: Supplementary Table S7.1–**Thermocycler conditions for the detection of PPV.

| **Steps** | **Temperature** | **Time** | **Cycle** |
| --- | --- | --- | --- |
| Reverse transcription | 45°C | 10 min | 1 |
| Initial denaturation | 95°C | 10 min | 1 |
| Denaturation | 94°C | 5 s | 45 |
| Annealing/Extension | 60°C | 1 min |  |
| Hold | 4°C | ∞ | **-** |

A multiplex RT-qPCR was performed using the TaqMan™ Fast Virus 1-Step Master Mix (ThermoFisher), in a total reaction volume of 20 µL containing 8 µL of template.

A recombinant plasmid containing the PPV NS1 gene fragment, as described by Shen [6] was used as a positive control.

Samples with a cycle threshold (*C*_t_) value below 37 were interpreted as positive. Samples with Ct ≥37 were considered inconclusive. If no amplification was detected, the sample was considered negative.

**Supplementary Material 9: Supplementary Table S8–**Thermocycler conditions for the detection of rotavirus (RVs) subtypes RVA, RVB, and RVC.

| **Steps** | **Temperature** | **Time** | **Cycle** |
| --- | --- | --- | --- |
| Reverse transcription | 50°C | 10 min | 1 |
| Initial denaturation | 95°C | 2 min | 1 |
| Denaturation | 95°C | 15 s | 40 |
| Annealing/Extension | 60°C | 30 s |  |
| Hold | 4°C | ∞ | **-** |

A one step multiplex real-time reverse transcription-PCR (RT-qPCR) was done using the VetMAX™ Rotavirus A/B/C Controls (Applied Biosystems, Thermo Fisher Scientific), which include validated primers and probes targeting rotavirus subtypes A, B, and C.

The RT-qPCR consisted of 20 µL containing 10 µL of 2X Master Mix, 5 μL of VetMAX Rotavirus Primer/Probe Mix, and 5 μL of template RNA. Fluorescent probes included ABY™, FAM™, and VIC™ dyes for RVA, RVB, and RVC, respectively and the Cy® 5 dyes for the Xeno internal positive control (IPC) RNA.

Samples with Ct < 35 for any target were considered positive. Samples without target amplification but with IPC amplification were considered negative. Samples lacking IPC amplification were retested to assess potential inhibition. No‑template controls (NTCs) showed no amplification.

**Supplementary Material 10: Supplementary Table S9–**Thermocycler conditions for the detection of porcine reproductive and respiratory syndrome virus (PRRSV).

| **Steps** | **Temperature** | **Time** | **Cycle** |
| --- | --- | --- | --- |
| Reverse transcription | 45°C | 10 min | 1 |
| Initial denaturation | 95°C | 10 min | 1 |
| Denaturation | 94°C | 5 s | 40 |
| Annealing/Extension | 60°C | 1 min |  |
| Hold | 4°C | ∞ | **-** |

A RT-qPCR was done using the VetMAX PRRSV 3.0 Reagents (ThermoFisher) as suggested by the manufacturer. PCR amplification and detection were done in a Light Cycler 480 (Roche). Samples with a Ct <36 were considered positive, and samples with Ct ≥36 were considered inconclusive. If no amplification was detected (e.g., no Ct value was produced), the sample was considered negative.

**Supplementary Material 11: Supplementary Table S10**–Summary of Alberta wild pigs tested for target pathogens across sampling years (2018–2024).

| **Pathogen** | **Year** | | | | | | | |
| --- | --- | --- | --- | --- | --- | --- | --- | --- |
|  | **2018** | **2019** | **2020** | **2021** | **2022** | **2023** | **2024** | **Overall** |
|  | **P% (NP/*n*)** | **P% (NP/*n*)** | **P% (NP/*n*)** | **P% (NP/*n*)** | **P% (NP/*n*)** | **P% (NP/*n*)** | **P% (NP/*n*)** | **P% (NP/*n*)** |
| **Serology** |  |  |  |  |  |  |  |  |
| *E. rhusiopathiae* | NT | NT | NT | 100% (61/61) | 100% (26/26) | 100% (9/9) | 100% (24/24) | 100% (120/120) |
| PCV2 | NT | NT | NT | 45.9% (28/61) | 3.9% (1/26) | 11.1% (1/9) | 33.3% (8/24) | 31.7% (38/120) |
| *Mhyo* | NT | NT | NT | 0% (0/61) | 7.7% (2/26) | 0% (0/9) | NT | 2.1% (2/96) |
| PRRSV type 1 (EU) | NT | NT | NT | 0% (0/61) | 3.84 (1/26) | 0% (0/9) | NT | 1.0% (1/96) |
| PRRSV type 2 (NA) | NT | NT | NT | 0% (0/61) | 0% (0/26) | 0% (0/9) | NT | 0% (0/96) |
| **Bacteriology** |  |  |  |  |  |  |  |  |
| *Salmonella* spp. | 0% (0/12) | 0% (0/6) | 0% (0/36) | 11.1% (2/18) | NT | NT | NT | 2.8% (2/72) |
| **Parasitology** |  |  |  |  |  |  |  |  |
| *Eimeria* spp. | 0% (0/8) | 43.3% (13/30) | NT | 49.0% (24/49) | 19.0% (11/58) | 15.4% (2/13) | 76.5% (26/34) | 39.6% (76/192) |
| *Metastrongylus* spp. | 0% (0/8) | 46.7% (14/30) | NT | 20.4% (10/49) | 17.2% (10/58) | 23.1% (3/13) | 35.3% (12/34) | 25.5% (49/192) |
| *Trichuris* spp. | 0% (0/8) | 13.3% (4/30) | NT | 6.12% (3/49) | 3.45% (2/58) | 0% (0/13) | 5.88% (2/34) | 5.7% (11/192) |
| *Ascaris* spp. | 0% (0/8) | 3.33% (1/30) | NT | 6.12% (3/49) | 3.45% (2/58) | 0% (0/13) | 14.7% (5/34) | 5.7% (11/192) |
| *Strongyloides* spp. | 0% (0/8) | 26.7% (8/30) | NT | 0% (0/49) | 0% (0/58) | 0% (0/13) | 0% (0/34) | 4.2% (8/192) |
| **Molecular** |  |  |  |  |  |  |  |  |
| PCV1 | 5.6% (1/18) | 0% (0/32) | 0% (0/14) | 0% (0/100) | 0% (0/79) | 0% (0/25) | 0% (0/63) | 0.3% (1/331) |
| PCV2 | 5.6% (1/18) | 0% (0/32) | 0% (0/14) | 0% (0/100) | 0% (0/79) | 0% (0/25) | 0% (0/63) | 32.9% (109/331) |
| PCV3 | 5.6% (1/18) | 0% (0/32) | 0% (0/14) | 0% (0/100) | 0% (0/79) | 0% (0/25) | 0% (0/63) | 27.2% (90/331) |
| *Salmonella* spp. | 0% (0/12) | 0% (0/6) | 0% (0/36) | 11.1% (2/18) | NT | NT | NT | 2.8% (2/72) |
| PPV | (0/1) | (2/4) | (7/12) | (2/39) | (0/10) | (0/6) | (7/29) | 17.8% (18/101) |
| PDCoV | 0% (0/18) | 0% (0/7) | 0% (0/36) | 0% (0/37) | NT | NT | NT | 0% (0/98) |
| PEDV | 0% (0/18) | 0% (0/7) | 0% (0/36) | 0% (0/37) | NT | NT | NT | 0% (0/98) |
| SVA | 0% (0/18) | 0% (0/7) | 0% (0/36) | 0% (0/35) | NT | NT | NT | 0% (0/96) |
| TGEV | 0% (0/18) | 0% (0/7) | 0% (0/36) | 0% (0/30) | NT | NT | NT | 0% (0/91) |
| IAV | 0% (0/1) | 0% (0/30) | 0% (0/1) | 0% (0/37) | 0% (0/8) | 0% (0/4) | 0% (0/2) | 0% (0/83) |
| RVA | NT | NT | NT | NT | 24.2% (8/33) | 18.8% (3/16) | 23.3% (10/43) | 22.8% (21/92) |
| RVB | NT | NT | NT | NT | 0% (0/33) | 0% (0/16) | 0% (0/43) | 0% (0/92) |
| RVC | NT | NT | NT | NT | 0% (0/33) | 31.3% (5/16) | 0% (0/43) | 5.4% (5/92) |

P%: Sample prevalence.

NP: Number of positive.

*n:* Total of samples tested.

NT: No test performed.

**Supplementary Material 12: Supplementary Table S11**–Post‑hoc minimum detectable prevalence estimates for pathogens tested. Minimum detectable prevalence was calculated assuming a 95% detection probability. Exact 95% binomial confidence intervals indicate the upper bound of prevalence compatible with zero positives.

| **Pathogen^a^** | **Tested (*n*)** | **Minimum detectable prevalence (%)** | **Exact binomial confidence** | |
| --- | --- | --- | --- | --- |
| **Serology** |  |  |  |  |
| *E. rhusiopathiae* | 120 | 2.47 | 0 – 3.03 |  |
| PCV2 | 120 | 2.47 | 0 – 3.03 |  |
| *Mhyo* | 96 | 3.07 | 0 – 3.77 |  |
| PRRSV type 1 (EU) | 96 | 3.07 | 0 – 3.77 |  |
| PRRSV type 2 (NA) | 96 | 3.07 | 0 – 3.77 |  |
| **Bacteriology** |  |  |  |  |
| *Salmonella* spp. | 72 | 4.08 | 0 – 4.99 |  |
| **Parasitology** |  |  |  |  |
| *Eimeria* spp. | 192 | 1.55 | 0 – 1.90 |  |
| *Metastrongylus* spp. | 192 | 1.55 | 0 – 1.90 |  |
| *Trichuris* spp. | 192 | 1.55 | 0 – 1.90 |  |
| *Ascaris* spp. | 192 | 1.55 | 0 – 1.90 |  |
| *Strongyloides* spp. | 192 | 1.55 | 0 – 1.90 |  |
| **Molecular** |  |  |  |  |
| PCV1 | 331 | 0.90 | 0 – 1.11 |  |
| PCV2 | 331 | 0.90 | 0 – 1.11 |  |
| PCV3 | 331 | 0.90 | 0 – 1.11 |  |
| *Salmonella* spp. | 72 | 4.08 | 0 – 4.99 |  |
| PPV | 101 | 2.92 | 0 – 3.59 |  |
| PDCoV | 98 | 3.01 | 0 – 3.69 |  |
| PEDV | 98 | 3.01 | 0 – 3.69 |  |
| SVA | 96 | 3.07 | 0 – 3.77 |  |
| TGEV | 91 | 3.24 | 0 – 3.97 |  |
| IAV | 83 | 3.54 | 0 – 4.35 |  |
| RVA | 92 | 3.20 | 0 – 3.93 |  |
| RVB | 92 | 3.20 | 0 – 3.93 |  |
| RVC | 92 | 3.20 | 0 – 3.93 |  |

**Supplementary Material 13: Supplementary Table S12**–Description and calculation of spatial covariates included in the mixed-effects logistic regression models.

| **Covariate** | **Description** | **Source** | **Calculation method** | **R package** |
| --- | --- | --- | --- | --- |
|  |  |  |  |  |
| Commercial pig density | Density of commercial pig farms (head/km^2^) | Government of Alberta | Kernel Density Estimation (KDE)* | spatialEco [7] |
| Small-scale pig density | Density of small -scale pig farms (head/km^2^) |  |  |  |
|  |  |  |  |  |
| Distance to commercial pig farms | Distance to nearest commercial pig farms (km) |  | Minimum Euclidian distance (ED) | sf [8] |
| Distance to small-scale pig farms | Distance to nearest small-scale pig farms (km) |  |  |  |
| Distance to historical wild pig farms | Distance to nearest historical wild pig farms (km) |  |  |  |

**Kernel bandwidth was selected using Scott's rule of thumb based on the spatial distribution of farm locations. Relative density surfaces were generated at a spatial resolution of 1 km.*

**Supplementary Material 14: Supplementary Table S13**–Univariate mixed-effects logistic regression analysis of risk factors (categorical predictors) associated with RVA-molecular detection in wild pigs captured in Alberta.

| **Variable** | **Category** | **Positive**  **(*n*=21)** | **Negative**  **(*n*=71)** | **ß ^a^** | **SE ^b^** | **OR ^c^** | **95% CI ^d^** | **P-value** |
| --- | --- | --- | --- | --- | --- | --- | --- | --- |
|  |  | **N (%)** | **N (%)** |  |  |  |  |  |
| **Sex (*n*=92)** | Female (*n*=39) | 11 (28.2) | 28 (71.8) | -0.899 | 0.451 | Ref | **-** | **-** |
|  | Male (*n*=53) | 10 (18.9) | 43 (81.1) | -0.653 | 0.569 | 0.52 | 0.17– 1.59 | 0.251 |
| **Age (*n*=92)** | Juvenile (*n*=11) | 2 (18.2) | 9 (81.8) | -0.522 | 1.263 | Ref | **-** | **-** |
|  | Mature (*n*=81) | 19 (23.5) | 62 (76.5) | -0.818 | 1.375 | 0.44 | 0.03-6.53 | 0.552 |
| **Pregnancy Status (*n*=37) ^e^** | No Pregnant (*n*=20) | 6 (30.0) | 14 (70.0) | -0.864 | 0.625 | Ref | **-** | **-** |
|  | Pregnant (*n*=17) | 5 (29.4) | 12 (70.6) | -0.189 | 0.883 | 0.83 | 0.15-4.67 | 0.830 |
| **County of captured**  **(*n*=92)** | LSA (*n*=32) | 5 (15.6) | 27 (84.4) | -1.704 | 0.597 | Ref | **-** | **-** |
|  | WL (*n*=39) | 11 (28.2) | 28 (71.8) | -0.506 | 1.283 | 0.60 | 0.05-7.45 | 0.693 |
|  | TH (*n*=11) | 4 (36.4) | 7 (63.6) | 1.115 | 0.965 | 3.05 | 0.46-20.2 | 0.248 |
|  | SC (*n*=10) | 1 (10.0) | 9 (90.0) | 0.752 | 0.723 | 2.12 | 0.51-8.79 | 0.300 |
| **Season of collection (*n*=92) ^f^** | Winter (*n*=77) | 20 (26.0) | 57 (74.0) | -1.004 | 0.349 | Ref | **-** | **-** |
|  | Spring + Summer (*n*=15) | 1 (6.7) | 14 (93.3) | -1.912 | 1.234 | 0.15 | 0.01-1.65 | 0.120 |
| **Year (*n*=92) ^g^** | 2022 (*n*=33) | 8 (24.2) | 25 (75.8) | -1.308 | 0.577 | Ref | **-** | **-** |
|  | 2023 (*n*=16) | 3 (18.8) | 13 (81.2) | -0.214 | 0.975 | 0.81 | 0.12-5.46 | 0.826 |
|  | 2024 (*n*=43) | 10 (23.3) | 33 (76.7) | 0.218 | 0.760 | 1.24 | 0.28-5.52 | 0.775 |

**^a^** ß: Estimate.

**^b^** SE: Standard Error.

**^c^** OR: Odds Ratio.

**^d^** CI: Confidence Interval.

**^e^** Data was not available for all individuals.

**^f^** Fall season was excluded because no samples were tested during that season. Spring and Summer were grouped to avoid sparse data and ensure stable model estimation.

**^g^** Analysis was restricted by year because testing began in 2022; no samples were tested for the period 2018-2021.

* Statistical Significance.

## Supplementary Material 14: Supplementary Table S13.1-Univariate mixed-effects logistic regression analysis of risk factors (continuous predictors) associated with RVA-molecular detection in wild pigs captured in Alberta.

| **Variable** | **ß ^a^** | **SE ^b^** | **OR ^c^** | **95% CI ^d^** | **P-value** |
| --- | --- | --- | --- | --- | --- |
|  |  |  |  |  |  |
| **Weight ^e^ (*n*=330)** | 0.006 | 0.012 | 0.99 | 0.97 – 1.02 | 0.596 |
| **Wild pigs group size (*n*=331)** | -0.091 | 0.045 | 0.91 | 0.84 – 1.00 | 0.043* |
| **Wild pig habitat**  **suitability (Index)** | -0.9120 | 2.376 | 0.40 | 0.00 – 42.3 | 0.701 |
| **Commercial pig**  **density (head/km^2^)** | -0.168 | 0.286 | 0.85 | 0.48 – 1.48 | 0.557 |
| **Small-scale pig density (head/km^2^)** | -0.381 | 0.236 | 0.68 | 0.43– 1.08 | 0.105 |
| **Distance to commercial pig farms (km)** | 0.005 | 0.034 | 1.01 | 0.94– 1.08 | 0.861 |
| **Distance to small-scale**  **pig farms (km)** | 0.315 | 0.183 | 1.37 | 0.96 – 1.96 | 0.085 |
| **Distance to wild**  **pig farms (km)** | 0.030 | 0.039 | 1.03 | 0.95 – 1.11 | 0.450 |

**^a^** ß: Estimate.

**^b^** SE: Standard Error.

**^c^** OR: Odds Ratio.

**^d^** CI: Confidence Interval.

**^e^** Data was not available for all individuals.

* Statistical Significance.

**Supplementary Material 15: Supplementary Table 14**–Univariate mixed-effects logistic regression analysis of risk factors (categorical predictors) associated with PPV-molecular detection in wild pigs captured in Alberta.

| **Variable** | **Category** | **Positive**  **(*n*=18)** | **Negative**  **(*n*=83)** | **ß ^a^** | **SE ^b^** | **OR ^c^** | **95% CI ^d^** | **P-value** |
| --- | --- | --- | --- | --- | --- | --- | --- | --- |
|  |  | **N (%)** | **N (%)** |  |  |  |  |  |
| **Sex (*n*=101)** | Female (*n*=51) | 7 (13.7) | 44 (86.3) | -2.450 | 0.733 | Ref | **-** | **-** |
|  | Male (*n*=50) | 11 (22.0) | 39 (78.0) | 0.542 | 0.664 | 1.72 | 0.47– 6.32 | 0.414 |
| **Age (*n*=101)** | Juvenile (*n*=21) | 2 (9.5) | 19 (90.5) | -3.792 | 1.371 | Ref | **-** | **-** |
|  | Mature (*n*=80) | 16 (20.0) | 64 (80.0) | 1.741 | 1.147 | 5.71 | 0.60-54.1 | 0.130 |
| **Season of collection (*n*=101)** | Fall (*n=*11) | 1 (9.1) | 10 (90.9) | -2.544 | 0.349 | Ref | **-** | **-** |
|  | Winter (*n*=55) | 8 (14.5) | 47 (85.5) | 0.640 | 1.394 | 1.90 | 0.12-29.2 | 0.646 |
|  | Spring (*n*=21) | 2 (9.5) | 19 (90.5) | 0.031 | 1.588 | 1.03 | 0.05-23.2 | 0.985 |
|  | Summer (*n*=14) | 7 (50.0) | 7 (50.0) | 2.093 | 1.590 | 8.11 | 0.36-183 | 0.188 |
| **Period (*n*=101)** | 2018-2021 (*n*=56) | 11 (19.6) | 45 (80.4) | -2.122 | 0.780 | Ref | **-** | **-** |
|  | 2022-2024 (*n*=45) | 7 (15.6) | 38 (84.4) | -0.058 | 0.913 | 0.94 | 0.16-5.65 | 0.949 |

**^a^** ß: Estimate.

**^b^** SE: Standard Error.

**^c^** OR: Odds Ratio.

**^d^** CI: Confidence Interval.

**^e^** Data was not available for all individuals.

Pregnancy status was excluded from the model because no positive cases occurred among pregnant females, preventing model convergence. County was excluded because all positive cases originated from a single county (WL), resulting in quasi‑complete separation.

* Statistical Significance.

## Supplementary Material 15: Supplementary Table 14.1–Univariate mixed-effects logistic regression analysis of risk factors (continuous predictors) associated with PPV-molecular detection in wild pigs captured in Alberta.

| **Variable** | **ß ^a^** | **SE ^b^** | **OR ^c^** | **95% CI ^d^** | **P-value** |
| --- | --- | --- | --- | --- | --- |
|  |  |  |  |  |  |
| **Weight ^e^ (*n*=330)** | 0.018 | 0.013 | 1.02 | 0.99 – 1.04 | 0.185 |
| **Wild pigs group size (*n*=331)** | -0.091 | 0.045 | 0.90 | 0.80 – 1.03 | 0.117 |
| **Commercial pig**  **density (head/km^2^)** | -0.690 | 0.787 | 0.50 | 0.11 – 2.35 | 0.382 |
| **Small-scale pig density (head/km^2^)** | -0.640 | 0.637 | 0.53 | 0.15– 1.84 | 0.315 |
| **Distance to commercial pig farms (km)** | 0.003 | 0.061 | 1.00 | 0.89– 1.13 | 0.995 |
| **Distance to small-scale**  **pig farms (km)** | 0.205 | 0.268 | 1.23 | 0.73 – 2.07 | 0.443 |
| **Distance to wild**  **pig farms (km)** | 0.037 | 0.089 | 0.96 | 0.81 – 1.15 | 0.681 |

**^a^** ß: Estimate.

**^b^** SE: Standard Error.

**^c^** OR: Odds Ratio.

**^d^** CI: Confidence Interval.

**^e^** Data was not available for all individuals.

* Statistical Significance.

**Supplementary Material 16: Supplementary Table 15**–Univariate mixed-effects logistic regression analysis of risk factors (categorical predictors) associated with *Eimeria* spp.-coprological detection in wild pigs captured in Alberta.

| **Variable** | **Category** | **Positive**  **(*n*=76)** | **Negative**  **(*n*=116)** | **ß ^a^** | **SE ^b^** | **OR ^c^** | **95% CI ^d^** | **P-value** |
| --- | --- | --- | --- | --- | --- | --- | --- | --- |
|  |  | **N (%)** | **N (%)** |  |  |  |  |  |
| **Sex (*n*=192)** | Female (*n*=94) | 37 (39.4) | 57 (60.6) | 0.597 | 0.233 | Ref | **-** | **-** |
|  | Male (*n*=98) | 39 (39.8) | 59 (60.2) | 1.050 | 0.379 | 1.05 | 0.51–2.13 | 0.903 |
| **Age (*n*=192)** | Juvenile (*n*=81) | 33 (40.7) | 48 (59.3) | 0.669 | 0.329 | Ref | **-** | **-** |
|  | Mature (*n*=111) | 43 (38.7) | 68 (61.3) | 0.889 | 0.439 | 0.89 | 0.34-2.34 | 0.813 |
| **Pregnancy Status (*n*=76) ^e^** | No Pregnant (*n*=56) | 22 (39.3) | 34 (60.7) | 0.660 | 0.259 | Ref | **-** | **-** |
|  | Pregnant (*n*=20) | 8 (40.0) | 12 (60.0) | 0.976 | 0.621 | 0.98 | 0.28-3.40 | 0.970 |
| **County of captured (*n*=192)** | LSA (*n*=47) | 18 (38.3) | 29 (61.7) | 0.348 | 0.250 | Ref | **-** | **-** |
|  | WL (*n*=124) | 49 (39.5) | 75 (60.5) | 2.140 | 1.770 | 2.14 | 0.42-10.8 | 0.425 |
|  | TH (*n*=15) | 6 (40.0) | 9 (60.0) | 1.190 | 1.460 | 1.19 | 0.11-13.2 | 0.108 |
| **Season of collection (*n*=192) ^f^** | SC (*n*=6) | 3 (50.0) | 3 (50.0) | 2.790 | 4.100 | 2.79 | 0.16-49.9 | 0.356 |
|  | Winter (*n*=89) | 34 (38.2) | 55 (61.8) | 0.751 | 0.310 | Ref | - | - |
|  | Spring + Summer (*n*=103) | 42 (40.8) | 61 (59.2) | 0.603 | 0.391 | 0.60 | 0.17-2.15 | 0.436 |
| **Period (*n*=192)** | 2018-2021 (*n*=87) | 37 (42.5) | 50 (57.5) | 0.413 | 0.228 | Ref | **-** | **-** |
|  | 2022-2024 (*n*=105) | 39 (37.1) | 66 (62.9) | 1.840 | 1.270 | 1.84 | 0.48-7.09 | 0.375 |

**^a^** ß: Estimate.

**^b^** SE: Standard Error.

**^c^** OR: Odds Ratio.

**^d^** CI: Confidence Interval.

**^e^** Data was not available for all individuals.

**^f^** Fall season was excluded because no samples were tested during that season. Spring and Summer were grouped to avoid sparse data and ensure stable model estimation.

* Statistical Significance.

## Supplementary Material 16: Supplementary Table 15.1–Univariate mixed-effects logistic regression analysis of risk factors (continuous predictors) associated with *Eimeria* spp.-coprological detection in wild pigs captured in Alberta.

| **Variable** | **ß ^a^** | **SE ^b^** | **OR ^c^** | **95% CI ^d^** | **P-value** |
| --- | --- | --- | --- | --- | --- |
|  |  |  |  |  |  |
| **Weight (*n*=192)** | -0.007 | 0.006 | 0.99 | 0.98 – 1.01 | 0.291 |
| **Wild pigs group size (*n*=192)** | 0.978 | 0.038 | 0.98 | 0.91 – 1.05 | 0.562 |
| **Commercial pig**  **density (head/km^2^)** | 0.960 | 0.312 | 0.96 | 0.51 – 1.81 | 0.901 |
| **Small-scale pig density (head/km^2^)** | 0.035 | 0.274 | 1.04 | 0.61– 1.77 | 0.897 |
| **Distance to commercial pig farms (km)** | 0.009 | 0.036 | 1.01 | 0.94– 1.08 | 0.781 |
| **Distance to small-scale**  **pig farms (km)** | 0.206 | 0.140 | 1.23 | 0.94– 162 | 0.139 |
| **Distance to wild**  **pig farms (km)** | -0.317 | 0.002 | 0.96 | 0.95 – 0.96 | 0.100 |

**^a^** ß: Estimate.

**^b^** SE: Standard Error.

**^c^** OR: Odds Ratio.

**^d^** CI: Confidence Interval.

Statistical Significance.

**Supplementary Material 17: Supplementary Table 16**–Univariate mixed-effects logistic regression analysis of risk factors (categorical predictors) associated with *Metastrongylus* spp.-coprological detection in wild pigs captured in Alberta.

| **Variable** | **Category** | **Positive**  **(*n*=76)** | **Negative**  **(*n*=116)** | **ß ^a^** | **SE ^b^** | **OR ^c^** | **95% CI ^d^** | **P-value** |
| --- | --- | --- | --- | --- | --- | --- | --- | --- |
|  |  | **N (%)** | **N (%)** |  |  |  |  |  |
| **Sex (*n*=192)** | Female (*n*=94) | 21 (22.3) | 73 (77.7) | -1.743 | 0.433 | Ref | **-** | **-** |
|  | Male (*n*=98) | 28 (28.6) | 70 (71.4) | 0.375 | 0.392 | 1.45 | 0.67–3.14 | 0.340 |
| **Age (*n*=192)** | Juvenile (*n*=81) | 24 (29.6) | 57 (70.4) | 0.213 | 0.121 | Ref | **-** | **-** |
|  | Mature (*n*=111) | 25 (22.5) | 86 (77.5) | 1.020 | 0.545 | 1.02 | 0.36-2.91 | 0.976 |
| **Pregnancy Status (*n*=76) ^e^** | No Pregnant (*n*=56) | 10 (17.9) | 46 (82.1) | 0.217 | 0.076 | Ref | **-** | **-** |
|  | Pregnant (*n*=20) | 4 (20.0) | 16 (80.0) | 1.150 | 0.758 | 0.98 | 0.28-3.40 | 0.832 |
| **County of captured (*n*=171) ^e, f^** | LSA (*n*=47) | 9 (19.1) | 38 (80.9) | -0.181 | 0.655 | Ref | **-** | **-** |
|  | WL (*n*=124) | 40 (32.3) | 84 (67.7) | 0.932 | 0.723 | 2.54 | 0.62-10.5 | 0.196 |
| **Season of collection (*n*=192)** | Fall (*n*=25) | 9 (36.0) | 16 (64.0) | -1.236 | 0.877 | Ref | - | - |
|  | Winter (*n*=64) | 18 (28.1) | 46 (71.9) | -0.459 | 0.184 | 0.96 | 0.15-6.24 | 0.963 |
|  | Summer (*n*=17) | 1 (5.9) | 16 (94.1) | -2.306 | 0.957 | 0.10 | 0.01-1.01 | 0.061 |
|  | Spring (*n*=86) | 21 (24.4) | 65 (75.6) | -0.459 | 0.391 | 0.63 | 0.09-4.41 | 0.643 |
| **Period (*n*=192)** | 2018-2021 (*n*=87) | 24 (27.6) | 63 (72.4) | -1.265 | 0.521 | Ref | **-** | **-** |
|  | 2022-2024 (*n*=105) | 25 (23.8) | 80 (76.2) | -0.446 | 0.671 | 0.64 | 0.17-2.38 | 0.505 |

**^a^** ß: Estimate.

**^b^** SE: Standard Error.

**^c^** OR: Odds Ratio.

**^d^** CI: Confidence Interval.

**^e^** Data was not available for all individuals.

**^f^** TH and SC county was excluded because all positive cases originated WL and LSA, resulting in quasi‑complete separation.

* Statistical Significance.

## Supplementary Material 17: Supplementary Table 16.1–Univariate mixed-effects logistic regression analysis of risk factors (continuous predictors) associated with *Metastrongylus* spp.-coprological detection in wild pigs captured in Alberta.

| **Variable** | **ß ^a^** | **SE ^b^** | **OR ^c^** | **95% CI ^d^** | **P-value** |
| --- | --- | --- | --- | --- | --- |
|  |  |  |  |  |  |
| **Weight (*n*=192)** | 0.004 | 0.007 | 1.00 | 0.99 – 1.02 | 0.953 |
| **Wild pigs group size (*n*=192)** | 0.040 | 0.038 | 1.04 | 0.97 – 1.12 | 0.283 |
| **Commercial pig**  **density (head/km^2^)** | -0.040 | 0.314 | 0.96 | 0.51 – 1.81 | 0.902 |
| **Small-scale pig density (head/km^2^)** | 0.156 | 0.299 | 1.17 | 0.65– 2.10 | 0.604 |
| **Distance to commercial pig farms (km)** | -0.086 | 0.045 | 0.92 | 0.84– 1.00 | 0.052 |
| **Distance to small-scale**  **pig farms (km)** | 0.029 | 0.159 | 1.03 | 0.75– 1.41 | 0.854 |
| **Distance to wild**  **pig farms (km)** | -0.157 | 0.066 | 0.85 | 0.75 – 0.97 | 0.017* |

**^a^** ß: Estimate.

**^b^** SE: Standard Error.

**^c^** OR: Odds Ratio.

**^d^** CI: Confidence Interval.

*Statistical Significance.

**Supplementary Material 18: Supplementary Fig. S1-** Parasite species identified in Alberta wild pigs from 2018-2024. (A, B) *Eimeria* spp. oocysts; (C, D) *Metastrongylus* spp. eggs; (E, F) *Trichuris* spp. eggs; and (G, H) *Ascaris* spp. eggs.

## Supplementary Material 19: Supplementary Statistical Analysis Methods References

- All the statistical analyses were performed with *R Software v4.5.2* [9]*.*
- Post hoc minimum detectable prevalence was estimated following the binomial detection‑probability framework described in [10], implemented in the *epiR* package [11]. Exact 95% binomial confidence intervals were calculated using the *binom* package [12].
- All data visualization was conducted using the *ggplot2* package [13].
- Pairwise pathogen associations were evaluated using the non‑parametric Phi coefficient test [14]. P‑values were adjusted using the Benjamini–Hochberg false discovery rate (FDR) method [15], and correlation matrices were generated with the *vcd* package [16].
- Univariate mixed‑effects logistic regression models were fitted using the *lme4* package [17], which implements generalized linear mixed models (GLMM). Model discrimination was assessed by calculating the Area Under the Receiver Operating Characteristic Curve (AUC‑ROC) using the *pROC* package [18].

## References

1. Gensler G, Pura J. Standard Operating Procedure: Real-time Polymerase chain reaction (RT-PCR) for detection of Salmonella species. 2018.

2. NCFAD. Standard Operating Procedure Approval: Real-Time Reverse Transcription Polymerase Chain Reaction for Detection of Seneca Valley Virus (SVV) Nucleic Acids. 2014.

3. Lu P, Lazaroff W. Standard Operating Procedure: Real-time Reverse Transcriptase Polymerase chain reaction (RRT-PCR) for detection of porcine coronaviruses. 2018.

4. CFIA. Detection of Type A Influenza Viruses and Avian H5 and H7 Hemagglutinin Subtypes by Real-Time RT-PCR Assay CFIA – NCFAD Controlled Document. 2020.

5. Palinski R, Piñeyro P, Shang P, Yuan F, Guo R, Fang Y, et al. A Novel Porcine Circovirus Distantly Related to Known Circoviruses Is Associated with Porcine Dermatitis and Nephropathy Syndrome and Reproductive Failure. J Virol. 2017;91:e01879-16. https://doi.org/10.1128/JVI.01879-16.

6. Shen HG, Beach NM, Huang YW, Halbur PG, Meng XJ, Opriessnig T. Comparison of commercial and experimental porcine circovirus type 2 (PCV2) vaccines using a triple challenge with PCV2, porcine reproductive and respiratory syndrome virus (PRRSV), and porcine parvovirus (PPV). Vaccine. 2010;28:5960–6. https://doi.org/10.1016/j.vaccine.2010.07.002.

7. Evans JS. spatialEco: Spatial Analysis and Modelling Utilities. CRAN: Contributed Packages. 2015. https://doi.org/10.32614/CRAN.package.spatialEco.

8. Pebesma E. sf: Simple Features for R. CRAN: Contributed Packages. 2016. https://doi.org/10.32614/CRAN.package.sf.

9. R Core Team. R: A Language and Environment for Statistical Computing. 2017.

10. Quach NE, Yang K, Chen R, Tu J, Xu M, Tu XM, et al. Post‐hoc power analysis: a conceptually valid approach for power based on observed study data. Gen Psychiatry. 2022;35:e100764. https://doi.org/10.1136/gpsych-2022-100764.

11. Stevenson M, Sergeant E. epiR: Tools for the Analysis of Epidemiological Data. 2025. https://doi.org/10.32614/CRAN.package.epiR.

12. Dorai-Raj. binom: Binomial Confidence Intervals for Several Parameterizations. CRAN: Contributed Packages. 2022. https://doi.org/10.32614/CRAN.package.binom.

13. Wickham H. ggplot2: Elegant Graphics for Data Analysis. 2016.

14. Chedzoy OB. Phi‐Coefficient. In: Encyclopedia of Statistical Sciences. Wiley; 2005. https://doi.org/10.1002/0471667196.ess1960.pub2.

15. Haynes W. Benjamini–Hochberg Method. New York, NY: Springer New York; 2013. https://doi.org/10.1007/978-1-4419-9863-7.

16. Meyer D, Zeileis A, Hornik K, Friendly M. vcd: Visualizing Categorical Data. 2024. https://doi.org/10.32614/CRAN.package.vcd.

17. Bates D, Mächler M, Bolker BM, Walker SC. Fitting Linear Mixed-Effects Models Using lme4. J Stat Softw. 2015;67:1–48. https://doi.org/10.18637/JSS.V067.I01.

18. Robin X, Turck N, Hainard A, Tiberti N, Lisacek F, Sanchez J-C, et al. pROC: an open-source package for R and S+ to analyze and compare ROC curves. BMC Bioinformatics. 2011;12:77. https://doi.org/10.1186/1471-2105-12-77.
